# A crucial RNA-binding lysine residue in the Nab3 RRM domain undergoes *SET1* and *SET3*-responsive methylation

**DOI:** 10.1093/nar/gkaa029

**Published:** 2020-01-21

**Authors:** Kwan Yin Lee, Anand Chopra, Giovanni L Burke, Ziyan Chen, Jack F Greenblatt, Kyle K Biggar, Marc D Meneghini

**Affiliations:** 1 Department of Molecular Genetics, University of Toronto, Toronto, ON M5G 1M1, Canada; 2 Institute of Biochemistry, Carleton University, Ottawa, ON K1S 5B6, Canada; 3 Donnelly Centre, University of Toronto, Toronto, ON M5S 3E1, Canada

## Abstract

The Nrd1–Nab3–Sen1 (NNS) complex integrates molecular cues to direct termination of noncoding transcription in budding yeast. NNS is positively regulated by histone methylation as well as through Nrd1 binding to the initiating form of RNA PolII. These cues collaborate with Nrd1 and Nab3 binding to target RNA sequences in nascent transcripts through their RRM RNA recognition motifs. In this study, we identify nine lysine residues distributed amongst Nrd1, Nab3 and Sen1 that are methylated, suggesting novel molecular inputs for NNS regulation. We identify mono-methylation of one these residues (Nab3-K363me1) as being partly dependent on the H3K4 methyltransferase, Set1, a known regulator of NNS function. Moreover, the accumulation of Nab3-K363me1 is essentially abolished in strains lacking *SET3*, a SET domain containing protein that is positively regulated by H3K4 methylation. Nab3-K363 resides within its RRM and physically contacts target RNA. Mutation of Nab3-K363 to arginine (Nab3-K363R) decreases RNA binding of the Nab3 RRM *in vitro* and causes transcription termination defects and slow growth. These findings identify *SET3* as a potential contextual regulator of Nab3 function through its role in methylation of Nab3-K363. Consistent with this hypothesis, we report that *SET3* exhibits genetic activation of *NAB3* that is observed in a sensitized context.

## INTRODUCTION

RNA PolII transcriptional termination is controlled through two distinct mechanisms in the budding yeast *Saccharomyces cerevisiae* (1–4). The first mechanism acts through the cleavage and polyadenylation factor (CPF), and couples the termination of protein encoding transcripts with their polyadenylation and nuclear export (5–7). The second termination mechanism functions through the Nrd1–Nab3–Sen1 (NNS) complex. NNS targets short noncoding RNAs such as snRNAs, snoRNAs and cryptic unstable transcripts (CUTS) for transcriptional termination, following which the transcripts are targeted for processing or degradation through the Exosome complex (8–11). NNS has a major role in the control of pervasive non-coding RNA transcription, which if left unregulated can interfere with the transcription of protein coding genes (8,9,11–13).

Multiple regulatory mechanisms function to restrict CPF and NNS termination to their respective target genes. CPF terminates at 3′ ends of genes through its recognition of poly-A signals in emerging RNA transcripts (6,14–16). CPF is further regulated through binding of its Pcf11 subunit to the serine-2 phosphorylated form of the PolII carboxy-terminal heptad repeat domain (CTD), which associates with transcriptional elongation (17–19). The action of CPF cleaves the elongating RNA transcript, leading to the processing of these nascent mRNAs for polyadenylation (20). In accordance with its action on short noncoding transcripts, NNS termination is promoted through interaction of the Nrd1 CTD interaction domain (CID) with the initiating/early-elongating form of RNA PolII, which exhibits the serine-5 phosphorylated form of CTD (19,21,22). Moreover, genetic evidence suggests that NNS is positively regulated through methylation of histone H3 on lysine-4 (H3K4me), a chromatin mark widely associated with transcriptional initiation and deposited by the conserved Set1 protein (23–30). By integrating these signals together with Nrd1 and Nab3 binding to cognate RNA sequences, NNS is thought to dislodge PolII from DNA in a manner that employs Sen1 ATPase activity (31–34).

Nab3 and Nrd1 RNA recognition sites are found broadly in the transcriptome (35). Moreover, the H3K4 methylation and PolII CTD serine-5 phosphorylation cues that promote NNS function are generic features of all PolII transcribed regions (21–24,36). As NNS does not act indiscriminately throughout the genome, it would seem that additional mechanisms must act to restrict NNS from inappropriate termination. Here we illuminate a potential new mode of NNS regulation through lysine methylation of its subunits. Using LC-MS/MS full scan, we identify nine lysine residues distributed amongst Nrd1, Nab3 and Sen1 that exhibit methylated forms. Many of these lysine residues are found within conspicuous protein domains of regulatory potential. We focus this study on the Nab3-K363 methylation site, which resides within the Nab3 RRM and is known to make contact with the RNA backbone of target transcripts (37,38). *NAB3* is an essential gene (39), and we find that mutation of Nab3-K363 to alanine (Nab3-K363A) leads to the production of a stable protein but nevertheless causes lethality. Mutation of Nab3-K363 to its most structurally similar residue arginine (Nab3-K363R) results in viable cells of greatly reduced health. The slow growth caused by Nab3-K363R is associated with transcription termination defects *in vivo* and reduced RNA binding affinity *in vitro*. Our findings reveal that the integrity of Nab3-K363 is crucial for NNS function because of its role in Nab3 RNA binding.

To shed light on the control of Nab3-K363 methylation, we considered the NNS regulator Set1 (23,24). Notably, we previously showed that *SET1* genetically opposes Nab3 function in a manner independent of H3K4 methylation, but dependent on a key catalytic residue of Set1 (24). This inhibitory impact of Set1 is counter-balanced by the known NNS-activating role of H3K4 methylation (23,24). Using SRM MS, we find that mono-methylated Nab3-K363 (Nab3-K363me1) is strongly reduced in *set1Δ*, suggesting that Set1 controls Nab3-K363 methylation either directly or indirectly in collaboration with one or more additional methyltransferases.

The Set3C histone deacetylase complex (HDAC) is positively regulated through binding to di-methylated H3K4 (H3K4me2) via the Set3 PHD domain (40). Interestingly, Set3, like its human ortholog MLL5, contains a SET domain potentially capable of lysine methyltransferase activity, though no confirmed substrate of the *SET3*/MLL5 family exists (41,42). Using SRM MS, we find that Nab3-K363me1 is essentially abolished in a strain lacking *SET3*. Sensitive genetic epistasis experiments reveal an activating role of *SET3* for *NAB3* that is only discerned in strains lacking *SET2*, which we also show here to repress *NAB3* function though its only known methylation target, H3K36. Our findings thus suggest a complexly acting, yet positive role for Nab3-K363me1. We discover and describe here a novel suite of lysine methylations on NNS, a crucial regulator of transcriptional termination in budding yeast. Our findings indicate that at least one of these methylations, Nab3-K363me1, participates in a complex network of NNS regulation involving H3K4 methylation, H3K36 methylation, Set1, Set2 and Set3. In addition to these new insights into NNS control, our findings identify the first lysine methylation reported to be responsive to the Set3/MLL5 family.

## MATERIALS AND METHODS

### Strains, media and plasmids

Standard *S. cerevisiae* genetic and strain manipulation techniques were used for strain construction and husbandry. All strains were constructed through crossing and dissection. Refer to Supplementary Table S1 for strains and plasmids used in this paper. *NAB3* and *NRD1* containing 600bp of flanking sequences were cloned into pRS313 and pRS316 using *in vivo* homologous recombination in yeast (43). Briefly, the parental plasmids were linearized with BamHI at the multiple cloning site and then transformed into budding yeast together with *NAB3* (600 bp up and down) or *NRD1* (600 bp up and down) containing 45 bp of homology to the BamHI cut site sequences produced by PCR amplification. Transformants that grew on -HIS dropout media (for pRS313) and -URA dropout media (for pRS316) were screened by PCR and verified by sequencing. All plasmids were tested for their ability to complement their respective deletion mutants. The pRS313-*SEN1* and pRS316-*SEN1* plasmids were acquired from Dr. David A. Brow (University of Wisconsin) (44). Q5 site-directed mutagenesis (NEB) was used to introduce nucleotide changes that translate to single amino acid substitutions into pRS313 plasmids harboring *NAB3*, *NRD1* and *SEN1*. For protein expression and purification from *Escherichia coli*, Nab3-RRM encoding sequences corresponding to residues 329–419 were amplified by PCR using the primer Nab3_329_forward 5′-GCATCATATGAAGTCAAGATTATTCATTGG-3′ and Nab3_419_reverse 5′-GCGCGGCCGCTTAAGTAGAACTACTGTTTGTACC-3′ from genomic *S. cerevisiae* DNA (37). After restriction digestion using NdeI and NotI (NEB), PCR products were ligated into the pET28a expression vector DNA resulting in an N-terminal fusion with a hexahistidine tag. Q5 site-directed mutagenesis (NEB) was used to introduce the K363R, K363A and S399A mutations into pET28a-NAB3-RRM (329–419). All plasmids were sequence verified.

### Serial dilution assay

Yeast strains were inoculated into several mL of -HIS -URA drop out media (YNB media (Multicell Wisent) containing 5 g/l of ammonium sulfate, -HIS -URA powder, 2% glucose) and grown overnight at 30°C. Each strain was diluted to an OD_600_ = 0.4, serially diluted five times, and spotted onto agar plates containing -HIS -URA dropout media 2% glucose, synthetic complete media 2% glucose supplemented with 0.1% 5-fluoroorotic acid (5FOA), or synthetic complete media as indicated.

### RNA extraction and RT-qPCR analysis

Strains were grown to mid-logarithmic phase and 5–10 OD_600_ equivalents of cells were harvested for RNA extraction. RNA was extracted with acidic phenol at 65°C for 30 min. RNA was then purified, precipitated, and resuspended in RNase free water. cDNA was prepared using either random nonamers or site-specific primers and Maxima H Minus Reverse Transcriptase (ThermoFisher Scientific) according to the manufacturer's instructions. PCR amplification of cDNA was detected using SYBR green in the BioRad iQ5 Multicolor Real Time PCR Detection System. All primer sequences are detailed in Supplementary Table S2. qPCR signal was normalized to the reference transcript *ACT1*. qPCR reactions were performed in triplicate per cDNA template and were carried out using the MyIQ5 real-time PCR detection system. The amount of qPCR signal was extracted using MyIQ5 software (BioRad). qPCR signal associated with the *ACT1* ORF was assessed in each biological replicate using cDNA generated by random priming. qPCR signal at different snoRNA loci and *NEL025C* was generated using the primers indicated in Figure 3 and Supplementary Table S2. For *SNR13*, cDNA was generated by *TRS31* gene specific priming or random priming. For other snoRNA loci and *NEL025C*, cDNA was generated by random priming. Relative read through was calculated by dividing qPCR signal at different snoRNA loci and *NEL025C* by *ACT1* levels in each biological replicate.

### Nab3-RRM purification

BL21(DE3) *E coli* cells containing pET28a-NAB3-RRM(329–419) (or mutant plasmids) were grown in 750 ml LB + 50 μg/ml kanamycin to OD_600_ = 0.4–0.6. Protein expression from pET28a plasmids was induced with 1 mM IPTG overnight at 16°C. Cells were then harvested into two 375 ml pellets stored in –80°C for future lysis. Nab3-RRM proteins were purified using Nickel Column Purification. Cell pellets were resuspended in PBS with protease inhibitors (E64, Bestatin, Pepstatin A, and PMSF), 1 mg/ml Lysozyme, 2% Triton X-100 and RNase-free DNaseI. Lysis was achieved by first sonicating for 4 cycles 30 s ON/OFF and then by incubating the cells for 20 min on a rotator at 37°C. Cell debris was clarified by centrifugation at 38 720 × g for 30 min. Cleared lysates were then passed through a Ni^2+^-NTA column (Qiagen 30250) that was equilibrated with P5 Buffer (20 mM NaHPO_4_ pH 7, 500 mM NaCl, 10% Glycerol, 0.05% Triton X-100, 5 mM Imidazole and 1 mM DTT). The column was then washed three times with P40 Wash Buffer (20 mM NaHPO_4_ pH 7, 500 mM NaCl, 10% glycerol, 0.05% Triton X-100, 40 mM Imidazole and 1 mM DTT). Elution was accomplished by passing 5 ml of P250 Elution Buffer (20 mM NaHPO_4_ pH 7, 500 mM NaCl, 10% glycerol, 0.05% Triton X-100, 250 mM Imidazole and 1 mM DTT) through the Ni^2+^-NTA column. Purified protein was dialyzed into buffer containing 20 mM Tris–HCl pH 8, 200 mM NaCl, 10% glycerol, 1 mM DTT and used for downstream applications. Protein concentration was determined by a standard Bradford assay.

### RNA binding assay

Purified hexahistidine-tagged Nab3 329–419 WT, K363R, K363A and S399A proteins were incubated with 25 μM biotinylated RNA probe (snR47; 5′-UUUCUUUUUUCUUAUUCUUAUU-3′) in binding buffer (10 mM Tris–HCl pH 7.8, 50 mM NaCl, 1 mM EDTA, 5% glycerol and 2.5 mM DTT) for 30 min at 15°C. Streptavidin–agarose beads (Novagen), blocked with bovine serum albumin, were added and rotated for 2 h at 4°C. Beads were washed four times with binding buffer containing 0.1% Tween-20 and bound proteins were eluted with 2× Laemmli Sample Buffer (Bio-Rad Laboratories). Eluted proteins were subjected to electrophoresis on a 17% resolving gel at 120 V for 2 h and transferred to PVDF membrane at 180 mA for 2 h and immunoblotted with His-Probe-HRP (Pierce) at a 1:5000 dilution. Immunoreactive bands were detected by chemiluminescence on a BioRad ChemiDoc XRS+ imager and quantified based on relative densitometry using ImageJ 1.51 v software. The equilibrium dissociation constant (*K*_d_) was determined based on densitometry of the visualized gel shift as previously published (45–47).

### Protein extraction and immunoblot analysis

One milliliter of logarithmically growing yeast cells was harvested for protein extraction. Cells were immediately pelleted and resuspended into 250 μl of 0.1 N NaOH for 5 min. The NaOH was then removed by centrifugation so that the cell pellet could be resuspended in 1× Laemmli Sample Buffer. This resuspension was boiled for 5 min. Total protein concentration was determined using an RC/DC assay (BioRad 5000121). Equal amounts of protein were electrophoresed on 8% or 10% SDS-PAGE gels and transferred onto Immobilon-PVDF Transfer membranes. (Millipore IPVH00010). Immunoblot analysis was performed using standard procedures. All blots were scanned with a BioRad ChemiDoc XRS+ Imaging System. Band intensities were quantified using ImageJ 1.51 v software.

Sen1 immunoblots were performed with slight differences from the standard protocol above as described previously (44). First, samples were electrophoresed using 4–15% Mini-PROTEAN TGX Precast Gels (Bio-Rad 4561086) at 140 V for 1 h transferred onto Immobilon-PVDF Membrane. Blots were blocked with 5% dried milk in TBST buffer with 0.1% Tween-20 at 23°C for 1 h, incubated with Sen1 antibody (1:2000 dilution) for 1 h and then an anti-rabbit secondary at 1:3000. Blots were scanned with a BioRad ChemiDoc XRS+ Imaging System and band intensities were quantified using ImageJ 1.51 v software.

### Immunoprecipitation

Co-immunoprecipitation was performed as described previously (48). Cell pellets containing 0.5 g of cells were collected from yeast cells grown to OD_6oo_ = 1–2. Pellets were frozen at –80°C until they were ready to be used. Frozen cell pellets were resuspended in Lysis Buffer (50 mM Na-HEPES pH 7.5, 200 mM NaOAc, pH 7.5, 1 mM EDTA, 1 mM EGTA, 5 mM MgOAc, 5% glycerol, 0.25% NP-40, 3 mM DTT, 1 mM PMSF and protease inhibitor cocktail (Roche 11836170001)) and lysed by three rounds of bead beating (BioSpec Mini-beadbeater-16), 1 min on and 1 min off on ice. Cell lysates were clarified by centrifugation. Total protein in the whole cell lysates was quantified using the RC/DC assay (BioRad 5000121). All samples were adjusted to the same concentration by adding Lysis Buffer. 30 μl of Protein A-Sepharose (15 μl of packed beads in a 50% slurry) was used per Anti-Nrd1 immunoprecipitation. 30 μl of Protein G-Sepharose (15 μl of packed beads in a 50% slurry) was used per Anti-Nab3 immunoprecipitation. Protein A-Sepharose beads were washed several times with Lysis Buffer and finally resuspended to 200 μl with 10 μl of anti-Nrd1 antibody. Protein G-Sepharose was put through the same protocol but with 5 μl of anti-Nab3 antibody. 500 μl of whole cell lysate was then added to the 200 μl of beads and antibody. Samples were placed on an end-over-end rotator at 4°C for 2 h. Beads were gently collected by low-speed centrifugation and wash several times using Wash Buffer (50 mM Na-HEPES pH 7.5, 200 mM NaOAc, pH 7.5, 1 mM EDTA, 1 mM EGTA, 5 mM MgOAc, 5% glycerol, 0.25% NP-40, 3 mM DTT and 1 mM PMSF). Samples were eluted by suspending beads in 50 μl 1× Laemmli Sample Buffer followed by a 65°C incubation for 10 min. Eluted proteins were immunoblotted for Nrd1, Nab3, Sen1 and Pgk1 as described above.

### Antibodies

An Anti-Nab3 (2F12) monoclonal mouse antibody was acquired from Dr. Maurice Swanson at the University of Florida and described previously (39). Anti-Nrd1 and anti-Sen1 rabbit antibodies were acquired from Dr David A. Brow at the University of Wisconsin (44). The Anti-Pgk1 antibody (ab113687) was purchased from Abcam. Antibodies were used at the following dilutions for immunoblot analysis: Nab3 (1:5000), Nrd1 (1:5000), Sen1 (1:5000) and Pgk1 (1:5000).

### TAP purification

Two step TAP purification was used to isolate Nrd1, Nab3 and Sen1 as previously described (49). Exponential cultures were grown overnight to OD_600_ between 1 and 2. Cells were harvested by centrifugation then washed once with cold water, once with cold Yeast Extract Buffer (YEB) (100 mM HEPES–KOH pH 7.9, 240 mM KCl, 5 mM EDTA, 5 mM (EGTA)–KOH pH 7.9 and 2.5 mM DTT), and once with cold YEB buffer containing protease inhibitor. For future use, cells were snap frozen in Falcon Tubes using liquid nitrogen. Cell lysis was performed by mechanically grinding the cell pellets into a fine powder with dry ice. The lysed powder was resuspended into an equal volume of YEB buffer containing protease inhibitor. Cell lysates were subject to ultracentrifugation to remove debris followed by dialysis to a buffer suitable for IgG binding (Dialysis buffer: 10 mM Tris–HCl pH 7.9, 100 mM NaCl, 0.2 mM EDTA, 0.5 mM DTT and 20% glycerol). TAP-tagged proteins were bound to IgG Sepharose beads that recognize the protein A moiety on the TAP tag. Protein associated with the IgG Sepharose beads were eluted using TEV protease, an enzyme that cleaves a linker region on the TAP tag, removing the protein A moiety permanently. In the second purification step, proteins that were freed from IgG were bound to Calmodulin Sepharose in a calcium dependent manner and subsequently eluted using EGTA. SDS-PAGE and silver staining were used to verify the presence of isolated protein complexes.

### Mass spectrometry

Samples were first digested using trypsin (Roche Diagnostics) overnight at 37°C. Digests were then desalted by C18-Zip Tip and dried in a SpeedVac. After desalting and drying, we reconstituted peptides in 20 μl of 0.1% FA and loaded 4 μl onto a Thermo Easy-Spray analytical column (75 μm i.d. × 500 mm) C18 column. Peptides were separated on a 125 min (5–40% acetonitrile) gradient. Mass spectra were collected on a Q-Exactive hybrid quadrupole-Orbitrap mass spectrometer coupled to an Easy-nLC 1000 system (ThermoFisher). The spectrometer was set in full MS/data-dependent-MS2 TopN mode: mass analyzer over *m*/*z* range of 400–1600 with a MS1 resolution of 70000 FWHM (at *m*/*z* = 200), 35 NEC (normalized collision energy), 2.0 *m*/*z* isolation window and 15 s dynamic exclusion. The MS2 data were obtained with a resolution of 35 000 FWHM. The PeaksX software (Bioinformatics Solutions Inc.) was used for data processing. Our methods used for the identification of Kme-modified peptides are similar to those previously described in Wang *et al.* (50). The spectra were first searched against the Uniprot/Swiss-prot protein database and filtered to 1% FDR (false discovery rate) for identification (Supplementary Figure S1). Default parameters were used with the following changes: peptides containing carbamidomethyl (C) as fixed modification, and oxidation (O), N-terminal acetylation (protein N-term), mono-methylation (K), di-methylation (K), tri-methylation (K) as variable modifications. Only tryptic peptides with up to two missed cleavage sites were allowed. Fragment mass tolerance was set to 15 ppm for Orbitrap MS2 with 0.5 Da for MS/MS fragment ions.

To further validate the Kme sites that were identified by the LC–MS/MS described above and explore the possibility of missed methylation states, we carried out targeted MS (i.e. SRM MS). For SRM MS, the *in silico* protease digest patterns and the corresponding transition ions were compiled with the Skyline software (51). Transitions that are larger than the precursor ion were selected on the basis of the Skyline predictions and the specific ions that allow unambiguous identification of the methylated lysine site and modification state were included. An isolation list was exported and used for the SRM MS method (Supplementary Table S3). Samples for SRM MS were digested and treated as mentioned above. The Skyline software was used for data processing.

To explore the influence of *SET1*, *SET2* or *SET3* expression over Nab3-K363 methylation, the same optimized isolation list (Supplementary Table S3) was also used to monitor relative site-specific methylation. Two-step TAP purified Nab3 protein from WT, *set1*Δ, *set2*Δ and *set3*Δ strains were digested and treated as mentioned above and subjected to SRM MS. All results were compiled by Skyline software. To account for minor deviations in peptide retention throughout the course of the study, all monitored peptides were normalized against the retention time of an internal control peptide in each experimental run (used as a relative zero point). For each sample, the total transition peak area for Nab3-K363me was used to determine relative methylation and was plotted against the WT strain. Relative transition peak area was then determined by dividing the total transition peak area of each *set*Δ strain to the total transition peak area of the WT strain.

## RESULTS

### Identification and characterization of NNS methylated lysine residues

**Figure 1.**
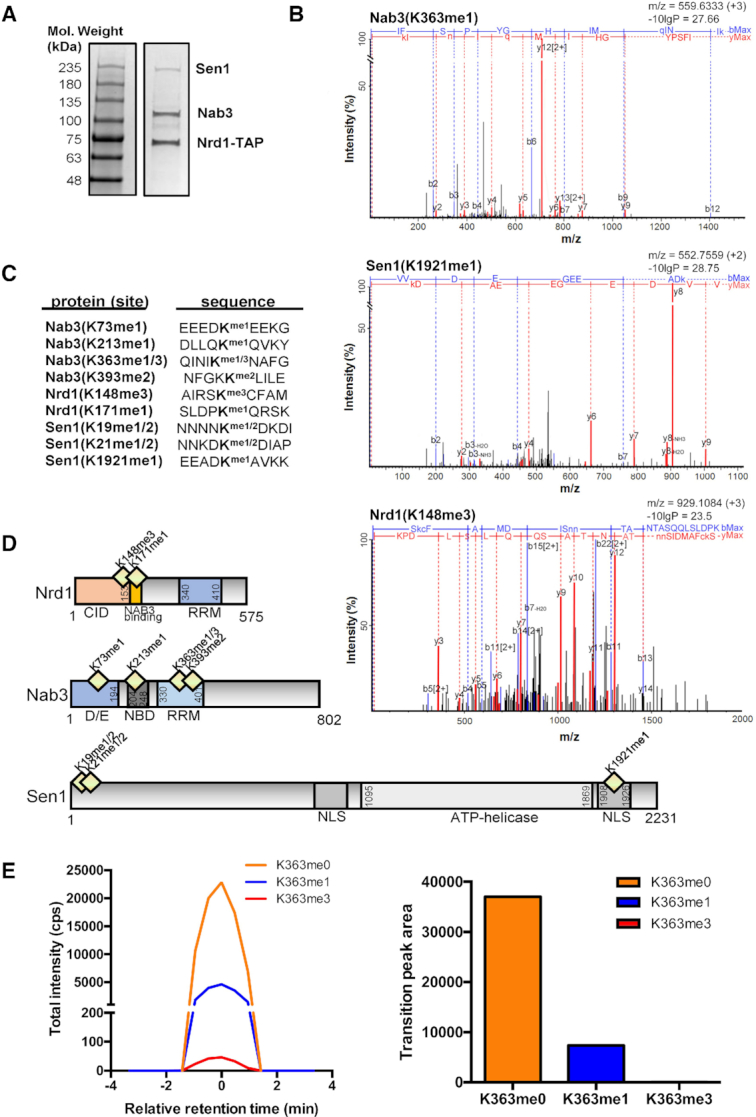
Multiple lysine residues on Nrd1, Nab3 and Sen1 are methylated. (**A**) The Nab3-Nrd1-Sen1 (NNS) complex was affinity purified through Nab3 tandem affinity purification. A Coomassie stained SDS-PAGE gel is shown. (**B**) Representative mass spectra for the identification of novel Nrd1, Nab3 and Sen1 lysine methylations are shown. Mass spectra for Nab3(K363me1), Nrd1(K148me1), and Sen1(K1921me1) are shown. Major y-ions are indicated as red peaks, while b-ions are blue. PEAKS peptide score (-10lgP) is shown for each mass spectrum. (**C**) A total of nine NNS methylation events were discovered by LC–MS/MS (QE Orbitrap) and post-analysis using PEAKS Studio software. (**D**) Nrd1, Nab3 and Sen1 lysine methylations mapped to functional domains of their respective proteins. (**E**) A chromatograph showing the relative retention time and peak intensities corresponding to Nab3-K363me0, Nab3-K363me1 and Nab3-K363me3 (left) and peak area quantification (right) is shown. Abbreviations: CID (CTD-interacting domain), D/E (D/E-rich), NBD (Nrd1 binding domain), RRM (RNA recognition motif), NLS (nuclear localization sequence).

We previously showed that NNS function was negatively regulated through one or more histone lysine methyltransferases in a manner independent of histone methylation (24). To investigate this, we used a proteomic approach to identify components of the NNS complex that undergo lysine methylation. NNS was purified by tandem affinity purification using Nab3-TAP (Figure 1A). The purified NNS complex was subjected to LC-MS/MS to facilitate the discovery of methylated lysines. Using this approach, we identified nine lysines distributed amongst the three subunits that were mono-, di-, or trimethylated (Figure 1B, C and Supplementary Figure S2). Many of these modified lysine residues map to functional domains of NNS subunits (Figure 1D). For example, Nab3-K213me1 maps within a domain of Nab3 that mediates its interaction with Nrd1 (Figure 1D) (10). Moreover, both K363me1 and K393me2 of Nab3 reside within its RRM (Figure 1D) (52). Lysine methylations on Nrd1 also map to functional domains (Figure 1D). Nrd1-K148me3 is located within its CID, while Nrd1-K171me1 maps to its Nab3 interaction domain (Figure 1D) (10). Three methylated lysine residues were identified within Sen1 (Figure 1C and D). Interestingly, both Sen1-K19 and Sen1-K21 exhibit both mono- and di-methylated forms (Figure 1C and D). Although the N-terminus of Sen1 possesses no predicted secondary structure, two-hybrid analysis identified physical interactions of this region with the Rad2 and Rnt1 proteins, which are involved in DNA repair and RNA processing (53). Sen1-K1921me1 resides within one of Sen1’s two functionally confirmed nuclear localization sequences (Figure 1D) (44,54).

**Figure 2.**
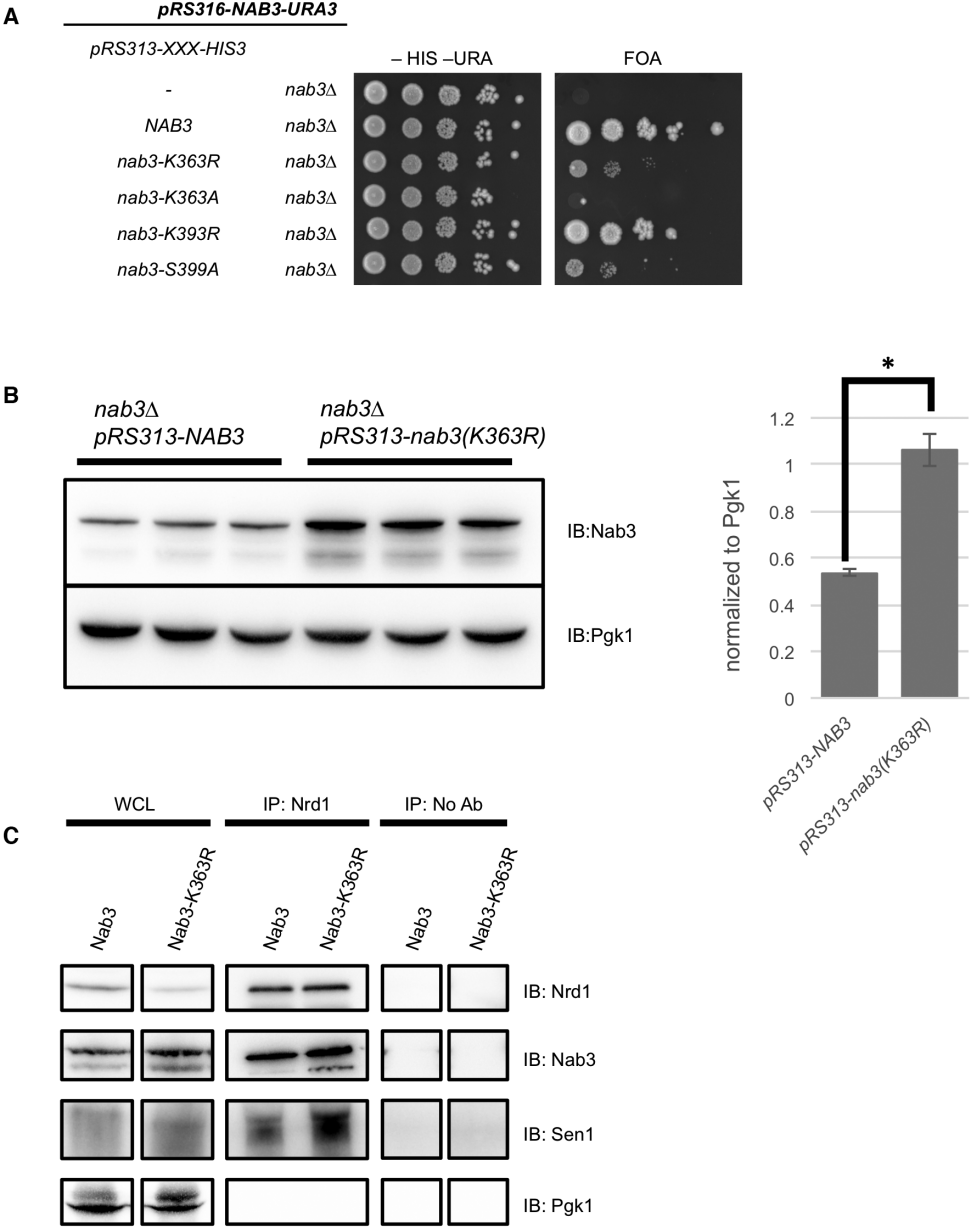
The Nab3 lysine 363 residue is important for cell viability. (**A**) Mutations in *NAB3* were introduced using a plasmid shuffling approach, in which a plasmid expressing a *NAB3* allele *(*pRS313-*nab3-allele-HIS3)* was transformed into a *nab3Δ* strain complemented with the pRS316-*NAB3-URA3* plasmid. Strains containing both plasmids were serially diluted 10-fold, spotted onto agar plates containing -HIS -URA dropout media and also on synthetic complete media supplemented with 5FOA to select against the pRS316-*NAB3-URA3* plasmid. From top to bottom: *pRS313-HIS3* (empty vector), *pRS313-NAB3-HIS3* (wildtype), *pRS313-nab3-K363R-HIS3, pRS313-nab3-K363A-HIS3, pRS313-nab3-K393R-HIS3*, and *pRS313-nab3-S399A-HIS*. K→R is a lysine to arginine substitution, K→A is a lysine to alanine substitution, S→A is a serine to alanine substitution. (**B**) Immunoblot analysis of Nab3 protein levels in wildtype and *nab3-K363R* mutants. Three biological replicates of each genotype are shown. Protein levels were quantified relative to the loading control Pgk1. Error bars represent standard deviation of three biological replicates, and significance was calculated using a two-tailed Student's *t*-test and denoted by *P < 0.01. (**C**) Nrd1 immunoprecipitation in wildtype and *nab3-K363R* mutants. Total cell lysates (WCL) were immunoprecipitated with a polyclonal Nrd1 antibody (IP: Nrd1) or no antibody (No Ab) as a negative control followed by immunoblot analysis with anti-Nrd1, anti-Nab3, anti-Sen1 and anti-Pgk1 antibodies.

To assess whether any of the lysine residues that undergo methylation exhibited a detectable role in NNS function, we mutated each residue to arginine (K→R) (Supplementary Figure S3). As the components of the NNS complex are all encoded by essential genes, mutant alleles of NNS were introduced into yeast strains using a plasmid shuffling approach (55). In this approach, the viability of *nrd1Δ*, *nab3Δ*, and *sen1Δ* strains depended on the presence of their respective wildtype genes on low-copy *URA3*-based vectors. Mutant alleles of *nrd1*, *nab3* and *sen1* were generated on a second low-copy *HIS3*-based vector and introduced into their respective deletion mutants. Thus, strains harboring both the *URA3* plasmid (wildtype) and the *HIS3* plasmid (mutant allele) were obtained. These strains were then spotted onto agar plates with -HIS -URA dropout media as a control and synthetic complete media containing 0.1% 5FOA to select against cells with the *URA3*-based vector. Cells grown on -HIS -URA dropout media maintain both vectors and all displayed comparable growth regardless of the contents of their *HIS3*-based vectors (Supplementary Figure S3). As expected, the *HIS3-*based empty vector did not support growth, but *nrd1Δ*, *nab3Δ* or *sen1Δ* were complemented with the *HIS3-*based vector containing their respective wildtype genes (Supplementary Figure S3). No growth defects were observed when Nrd1 and Sen1 lysine methylation sites were mutated to arginine (Supplementary Figure S3A and C). For Nab3, we observed a severe growth defect when lysine-363 was mutated to arginine (Nab3-K363R), but no apparent phenotype for the Nab3-K73R, K213R, or K393R mutants (Figure 2A and Supplementary Figure S3). The Nab3-K363R phenotype was similarly manifested when all four methylated lysines were mutated to arginine (Supplementary Figure S3B). Western blotting revealed that all lysine to arginine mutant proteins were expressed stably (Figure 2B and Supplementary Figure S4).

To more sensitively interrogate the K→R mutants, we also tested if they affected the growth of temperature sensitive mutants of other NNS subunits in *trans* (for instance a *nab3-K73R* mutant in a *nrd1-102* mutant or a *sen1-1* mutant). Sen1 K→R mutants did not affect the growth of *nab3-11* or *nrd1-102* mutants; Nab3 K→R mutants did not affect the growth of *nrd1-102* or *sen1-1* mutants; and Nrd1 K→R mutants did not affect the growth of *nab3-11* or *sen1-1* mutants (data not shown). The only other phenotype we observed was a subtle but highly reproducible suppression of the *nrd1-102* allele by a cis mutation of K171 to arginine (Supplementary Figure S5). This suggests that Nrd1-K171, and perhaps methylation of Nrd1-K171, had some repressive consequence for Nrd1 function.

### Nab3-K363R encoded a stable protein that was assembled into NNS

Of the nine methylated lysine residues we identified, only mutation of Nab3-K363 resulted in a strong growth phenotype (Figure 2A and Supplementary Figure S3B). This result illuminated the significance of Nab3-K363 for its *in vivo* function, and we chose to focus on this residue for continued studies. To fully characterize the methylation profile of the Nab3-K363 site, we used SRM MS with an independently purified NNS complex to monitor its methylation status in a highly sensitive and targeted MS manner (Figure 1E). Using this approach, we found that Nab3-K363 existed in mono and tri-methylated forms; although Nab3-K363me1 exhibited approximately 100-fold increased detectability over Nab3-K363me3, suggesting that Nab3-K363me1 was of substantially greater abundance than Nab3-K363me3 (Figure 1E). Moreover, the unmethylated form of Nab3-K363 (Nab3-K363me0) was ∼4-fold more detectable than Nab3-K363me1, suggesting that Nab3-K363me1 occurred on a minority of Nab3 proteins (Figure 1E). The *nab3-K363R* mutant is similar to the well-studied *nab3-11* mutant in that both contain mutations in the RRM (F371L and P374L mutations for *nab3-11*) that cause slow growth and even temperature sensitivity for *nab3-11* (56). To highlight that the *nab3-K363R* phenotype was not a generic consequence of mutating any RRM residue, we note that the RRM associated Nab3-K393R mutant displayed wildtype-comparable growth (Figure 2A and Supplementary Figure S3B).

An explanation for the Nab3-K363R growth defect could be that it caused destabilization of the essential Nab3 protein. To test this, we measured Nab3 protein levels using immunoblotting. Surprisingly, we found that Nab3-K363R proteins levels were approximately two-fold higher than the wildtype Nab3 (Figure 2B and Supplementary Figure S4). This increase in Nab3-K363R protein levels was not due to an increase in mRNA abundance as *nab3-K363R* and *NAB3* transcript levels were equivalent (Supplementary Figure S6). Next, we asked if Nab3-K363R was incorporated into NNS, positing that improper formation of the NNS complex might cause the severe cell growth defect caused by *nab3-K363R*. To test the integrity of the NNS complex in wildtype and *nab3-K363R* mutants, we used a polyclonal antibody to immunoprecipitate Nrd1. Immunoblotting was then used to assess the levels of associated Nab3 and Sen1 in these immunoprecipitates. We found that Nrd1 robustly associated with Sen1 and Nab3 in WT and *nab3-K363R* strains (Figure 2C). In fact, Sen1 appeared to show modestly enhanced NNS association in the *nab3-K363R* strain, perhaps suggesting that Nab3-K363R somehow promoted Sen1 association, though this assay is only a semi-quantitative representation of the NNS interaction. A reciprocal immunoprecipitation was performed using a monoclonal Nab3 antibody, validating the interaction of Nrd1 with both wildtype Nab3 and Nab3-K363R (Supplementary Figure S7).

These findings suggest that the integrity of Nab3-K363 was of crucial importance for Nab3 function, and that was not due to any deficiency in NNS assembly. The K→R substitution was chosen as a conservative change using the rationalization that both lysine and arginine are positively charged amino acids with sidechains that have similar structures. To further investigate if the chemical properties of Nab3-K363 were important for its function, we constructed a Nab3-K363 to alanine (K→A) mutant. The sidechains of lysine and alanine are completely different both in structure and chemical properties. Although *nab3-K363A* encoded a stable protein, a *nab3Δ* strain complemented with a plasmid expressing *nab3-K363A* was inviable (Figure 2A and Supplementary Figure S8). Collectively, these findings show that the precise biochemical characteristics of Nab3-K363 were crucial for proper NNS function, and that this was unrelated to protein stability or complex formation.

### Nab3-K363R caused transcription read-through defects

**Figure 3.**
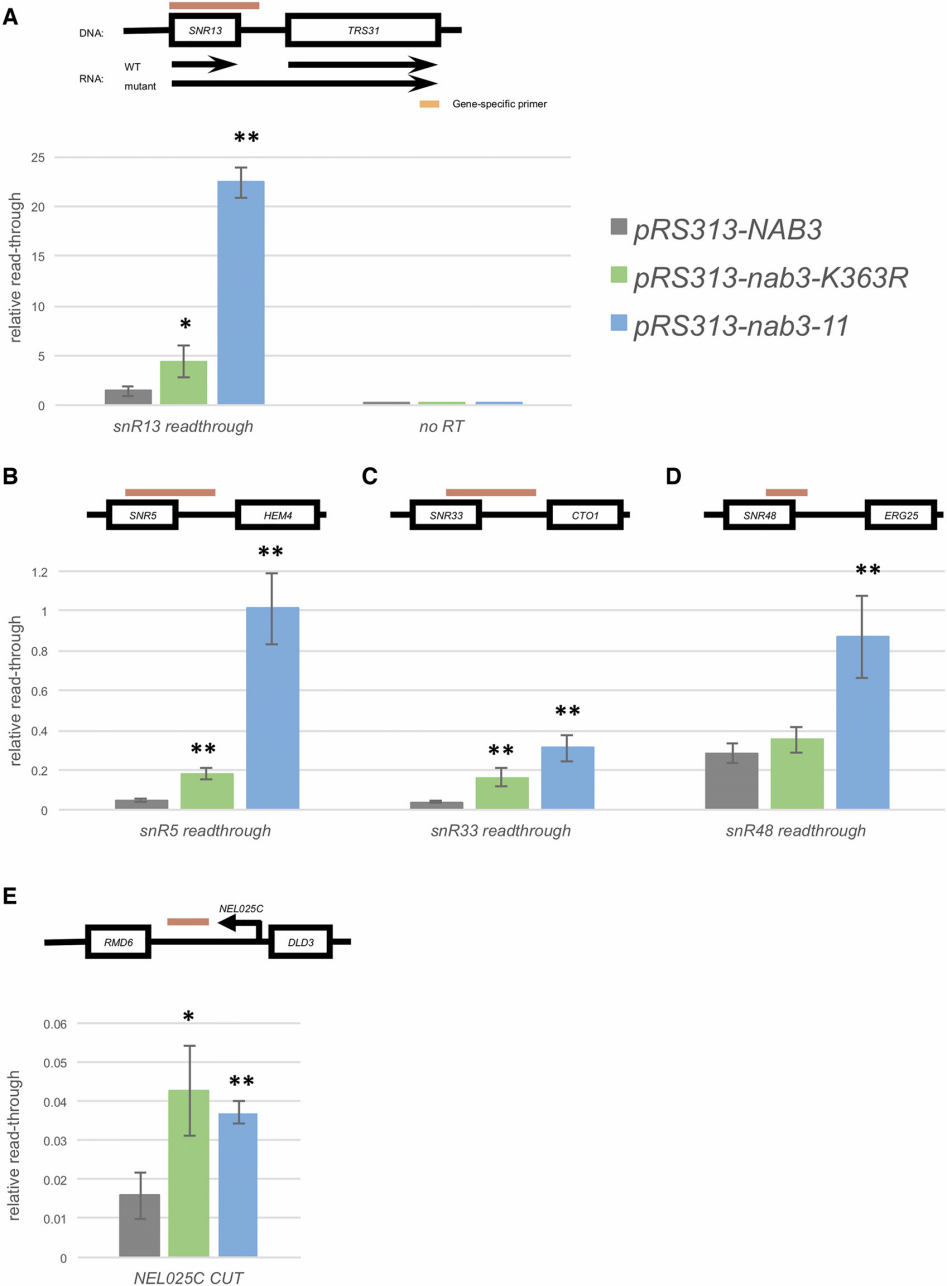
The Nab3-K363R mutant exhibits transcription termination defects at several noncoding RNAs. (**A**) Total RNA from *NAB3*, *nab3-K363R*, and *nab3-11* cells was processed into cDNA using a *TRS31* gene specific primer (illustrated as an orange rectangle). The presence of *SNR13* read-through transcription was analyzed by qPCR using a forward primer that anneals within the snoRNA and a reverse primer that anneals at a downstream intergenic region. The qPCR product is represented by the red rectangle. (**B–E**) Total RNA from *NAB3, nab3-K363R* and *nab3-11* cells was processed into cDNA using random nonamers. Read-through transcription was measured at the following snoRNA genes: (B) *SNR5*, (C) *SNR33*, (D) *SNR48*. RT-qPCR was also used to measure levels of the (E) *NEL025c CUT*. qPCR signal from the read-through and *NEL025c* primers was normalized to *ACT1* mRNA levels, which appear comparable in all three strains. Error bars represent standard deviation of three biological replicates. Significance between each mutant and the WT control (*pRS313-NAB3)* is calculated by a two-tailed Student's *t*-test and denoted by *P < 0.05, **P < 0.01. No reverse transcription control (No RT, *n* = 1).

Our findings implied that, despite permitting the assembly of an NNS complex, Nab3-K363R caused reduced NNS function. We evaluated this by investigating termination defects caused by Nab3-K363R. The *SNR13-TRS31* locus is commonly investigated for evidence of transcription read-through defects caused by reduced NNS function. *SNR13* encodes one of many snoRNA transcripts that are normally terminated by NNS (9,57). In NNS mutants, failed termination at *SNR13* leads to a read-through transcription through the downstream *TRS31* gene, which is terminated by CPF resulting in a concatenated *SNR13-TRS31* transcript (9). To investigate whether *nab3-K363R* mutants exhibited defects in transcription termination, we measured transcription read-through at *SNR13-TRS31* using a RT-qPCR approach. Initially, we used a *TRS31* gene specific primer to create cDNA from RNA isolated from wildtype, *nab3-K363R* and a *nab3-11* control. With this strategy, wildtype cells should yield a cDNA corresponding to the *TRS31* mRNA and no cDNA corresponding to *SNR13-TRS31* concatenated transcripts (Figure 3A). In contrast cells lacking proper NNS function should contain significantly more *SNR13-TRS31* transcripts (Figure 3A). The amount of *SNR13-TRS31* cDNA was determined with quantitative polymerase chain reaction (qPCR) using a forward primer that anneals within *SNR13* and a reverse primer that anneals in the intergenic region between *SNR13* and *TRS31* (Figure 3A). The level of read-through transcripts in each strain was normalized to *ACT1* mRNA levels. In agreement with previous studies, we found that strains with the *nab3-11* temperature sensitive allele accumulated significant levels of read-through transcripts at *SNR13-TRS31*, about 15-fold more read-through transcripts compared to wildtype (Figure 3A). In comparison, strains with the *nab3-K363R* allele accumulated three-fold more read-through transcripts than wildtype (Figure 3A).

To assess accumulation of read-through transcripts at a wide range of loci, we subsequently tested a random primer approach to create cDNA. We measured the levels of *SNR13-TRS31* cDNA generated by random priming using the same qPCR primers as above. Data from gene specific priming and random priming were comparable, with a 10-fold and 2.5-fold more read-through transcript accumulation compared to wildtype in the *nab3-11* and *nab3-K363R* mutants respectively (Supplementary Figure S9A). We similarly measured read-through transcript accumulation at a number of different snoRNA loci (Figure 3B–D and Supplementary Figure S9B). Generally, read-through transcripts accumulate to higher levels in *nab3-11* strains compared with *nab3-K363R* strains. For instance, *snR5-HEM4* and *SNR33-CTO1* loci follow the same trends as at *SNR13-TRS31*, with a significant accumulation of read-through transcripts in *nab3-K363R* and *nab3-11* compared to wildtype (Figure 3B and C). Interestingly, at *SNR47-YDR042C* and *SNR48-ERG25*, we found that read-through transcript accumulation was elevated in *nab3-11* but not *nab3-K363R* (Figure 3D and Supplementary Figure S9B). We also examined the cryptic unstable transcript *NEL025C*, which is terminated by NNS, leading to its instability (11). In contrast to the snoRNA read-through transcripts, *NEL025C* CUT accumulated to similar levels in both the *nab3-11* and *nab3-K363R* mutants (Figure 3E). These results suggest that Nab3-K363R caused widespread NNS transcriptional termination defects.

### Nab3-K363R caused reduced RNA binding *in vitro*

**Figure 4.**
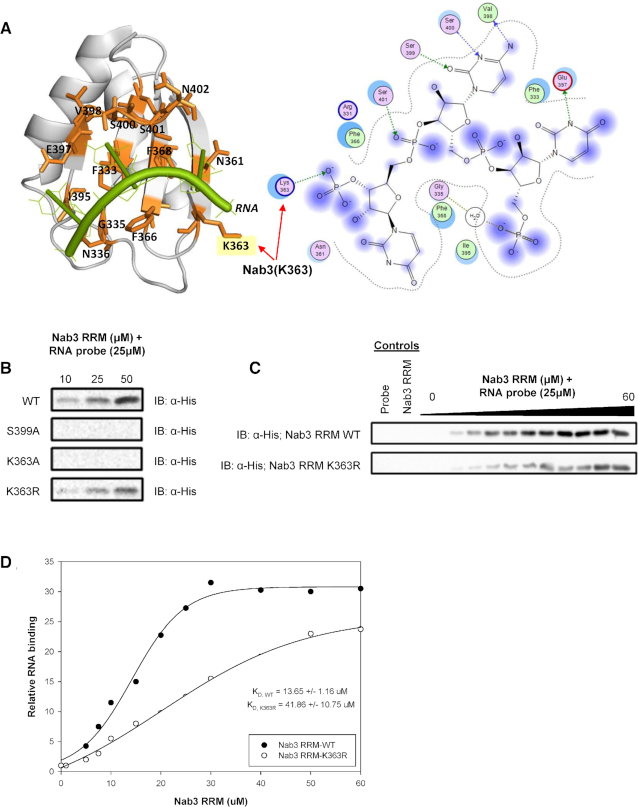
The Nab3 lysine-363 to arginine mutation attenuates RNA binding *in vitro*. (**A**) Co-crystal structure of Nab3 demonstrating the proximity (left) and interaction (right) of the K363 methylation site with bound RNA (PDB 2XNQ). Structures were visualized using PyMol (left) and Molecular Operating Environment (right). (**B**) Effect of single mutants (S399A, K363A, K363R) on RNA binding activity of Nab3 RRM (329–419) protein to 25 μM snR47 RNA probe (UUUCUUUUUUCUUAUUCUUAUU). Immunoblot detection by terminal 6xHis tag present on all Nab3 constructs. (**C**) Dose-response (0–60μM) of Nab3 RRM (329–419) and Nab3 RRM (329–419; K363R) to 25μM snR47 RNA probe. (**D**) Quantification of Nab3 RRM binding activity to snR47 probe. All RNA binding was monitored by RNA pull-down assay.

The conspicuous location of Nab3-K363 within its RRM suggested that this residue might be important for RNA binding. Indeed, X-ray crystallographic and NMR spectroscopy studies of Nab3-RRM bound to its UCUU recognition sequence revealed that Nab3-K363 makes contact with the RNA backbone (37,38). Specifically, the side-chain of Nab3-K363 forms a hydrogen bond with one of the bridging phosphates of the RNA (Figure 4A) (37,38). These previous findings led us to hypothesize that Nab3-K363R weakened the binding of Nab3 to its cognate RNA sequence. We tested this hypothesis using *in vitro* RNA pull-down assays, measuring the ability of bacterially expressed and purified wildtype and mutant Nab3 RRM proteins to bind to a biotinylated RNA probe corresponding to snR47. As expected, Nab3-RRM bound the probe robustly (Figure 4B–D). In agreement with a previous study, we did not detect RNA binding by a S399A RRM mutant protein, establishing the validity of our RNA binding assay (Figure 4B) (38). Intriguingly, although the Nab3 S399A mutant was previously reported to cause lethality in yeast (38), we were able to recover these mutants, though they were very slow growing (Figure 2A). Like with *nab3-K363A* and *nab3-K363R*, the *nab3-S399A* growth defect was not due to impaired protein expression (Supplementary Figure S8).

Next, we assessed Nab3-K363A and -K363R RRMs for their abilities to bind snR47 RNA. Like with the S399A mutant, we found that the K363A RRM also exhibited no RNA binding activity *in vitro* (Figure 4B). In contrast, the K363R RRM maintained the ability the bind RNA (Figure 4B), but at a significantly decreased affinity. We quantified this decreased binding by calculating the equilibrium dissociation constants (*K*_d_) for the wildtype and K363R Nab3 RRMs. Nab3-RRM binds the snR47 RNA with moderate affinity, exhibiting an apparent *K*_d_ of 13.65 μM (Figure 4C and D). The K363R mutation decreased snR47 RNA binding affinity of the Nab3 RRM by 3-fold (*K*_d_ of 41.86 μM). The differences in viability of wildtype versus *nab3-K363A* and *nab3-K363R* strains thus mirror their respective RNA binding capacity, revealing that the contacts between Nab3-K363 and the RNA backbone represent an important binding interface controlling essential NNS function. As such, a provocative interpretation of our findings is that methylation of Nab3-K363 influences its RNA binding affinity, though our results do not distinguish between potential positive or negative impacts of Nab3-K363me1.

### *SET1* and *SET3*-responsive methylation of Nab3-K363me1

**Figure 5.**
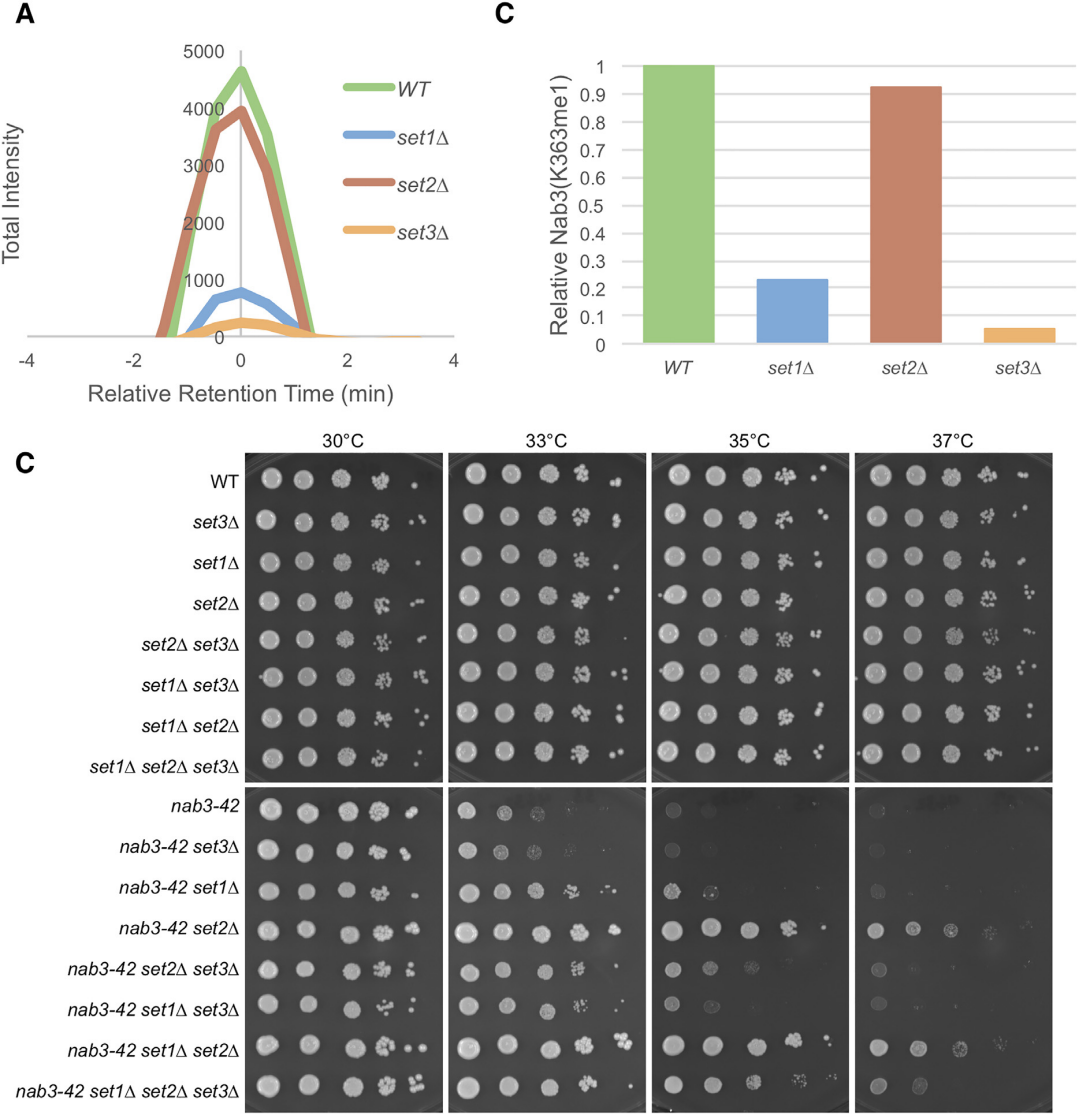
*SET1* and *SET3* control Nab3-K363me1 accumulation. (**A**) A chromatograph showing the relative retention time corresponding to Nab3-K363me1 in NNS complexes purified from WT versus *set1Δ*, *set2Δ* or *set3Δ* strains is shown. (**B**) Relative total peak area of Nab3-K363me1 chromatographs in WT versus *set1Δ*, *set2Δ* or *set3Δ* strains. (**C**) Strains of the indicated genotypes were serially diluted ten-fold and spotted onto synthetic complete plates and grown at 30°C at the shown temperatures.

A prerequisite necessary to understand the functional significance of Nab3-K363me is the identification of the methyltransferase(s) required for this modification. Previously, we reported that *SET1* repressed Nab3 function in a manner independent of H3K4 methylation but dependent on its methyltransferase activity, and that this repressive function was counterbalanced by an activating role for H3K4me3 (24). We note that studies in yeast have shown that, in addition to its well-known histone H3K4 substrate, Set1 also controls the methylation of Dam1, an essential kinetochore protein (58,59). This suggests that a potential mechanism through which Set1 lysine methyltransferase activity represses NNS could be through controlling a dynamic methylation of Nab3-K363. To test this hypothesis, we utilized SRM MS to monitor the relative site-specific changes in Nab3-K363 methylation levels in a highly-sensitive and targeted manner between WT and *set1Δ* strains. Relative Nab3-K363me levels were determined by comparing the total transition peak area for each strain (Figure 5A). We found that Nab3-K363me1 abundance decreased >4-fold in the *set1Δ* strain (Figure 5A and B). Although no differences were observed in Nab3-K363me3, the fleeting detectability of this modification state renders it difficult to precisely monitor (Figure 1E and data not shown). The difference in relative Nab3-K363me1 levels demonstrates a dependence of Nab3-K363me1 accumulation on the presence of *SET1*. A parallel analysis of the H3K36 methyltransferase, *SET2*, revealed essentially no difference in Nab3-K363me1 levels in a *set2Δ* mutant, highlighting the specificity of the *SET1* result (Figure 5A and B).

Set1 positively regulates the Set3C HDAC via its production of H3K4me2, which serves as a binding target for the Set3 PHD domain (40). We thus considered Set3 as a candidate protein that may accomplish Nab3-K363me1 in response to Set1/H3K4me-activation. We again used SRM MS to monitor relative Nab3-K363me1 changes in WT and *set3Δ* strains and observed a > 95% reduction in the mutant strain (Figure 5A and B). Together, these findings identify Set1 and Set3 as crucial mediators of Nab3-K363me1 accumulation, raising numerous potential regulatory scenarios, both direct and indirect, by which these proteins may promote Nab3-K363me1. To provide further confirmation of the specificity of these *SET1* and *SET3* effects, we interrogated each of the additional Nab3 and Sen1 methyl-lysine sites we discovered and found them to be relatively unperturbed in *set1Δ*, *set2Δ* and *set3Δ* strains (Supplementary Figure S10).

### Complex *NAB3* regulation by *SET1*, *SET2*, and *SET3*

**Figure 6.**
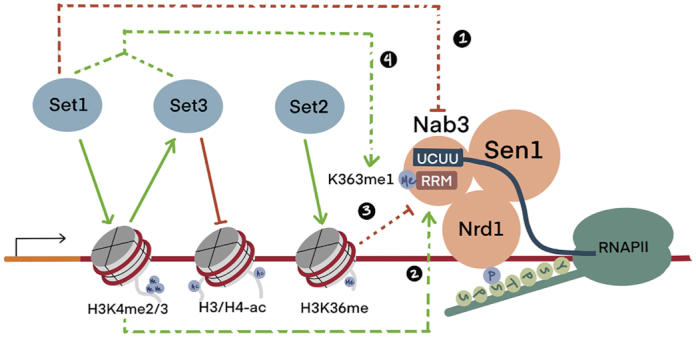
A complex chromatin nexus controls NNS. Solid lines show the known chromatin regulatory mechanisms of Set1, Set2 and Set3. Dotted lines represent genetic pathways of Nab3-NNS regulation and Nab3-K363me1 accumulation characterized here and elsewhere. (1) and (2) depict counterbalancing NNS control by Set1 through H3K4me independent and dependent mechanisms. (3) Illustrates Set2 repression of *NAB3* through H3K36me. (4) displays *SET1* and *SET3*-dependent Nab3-K363me1 accumulation. These mechanisms collaborate with Nrd1 binding to the RNA PolII CTD phosphorylated on serine-5.

To examine the potential regulatory role of Nab3-K363me1, we genetically interrogated interactions of *SET1*, *SET2* and *SET3* with *NAB3*. We found that the severe slow growth caused by Nab3-K363R was not altered by deletions of *SET1* or *SET3*, suggesting that mutation of this critical residue swamped out the ability to evaluate Nab3 regulation by these genes (data not shown). To assess the relative regulatory roles of Set1, Set2 and Set3 on Nab3 using a more sensitive approach, we evaluated genetic interactions of deletions of their respective genes when combined with temperature sensitive alleles of *NAB3* at semi-permissive temperatures. As we showed previously (24), *set1Δ* suppressed the growth defect of a *nab3-42* strain (Figure 5C). To our surprise, deletion of *SET2* provided substantially stronger suppression of *nab3-42* than *set1Δ*, and a *set1Δ set2Δ* mutant did not exhibit any synergy (Figure 5C). To determine if Set2 influenced *NAB3* through its only known substrate, H3K36, we generated strains expressing H3K36R in place of H3K36, and found that H3K36R phenocopied *set2Δ*, suggesting that a methylated form of H3K36 accounted for the repressive impact that *SET2* had on *NAB3* (Supplementary Figure S11). While deletion of *SET3* did not alter the growth of *nab3-42* on its own, *set3Δ* strongly reverted the suppression of *nab3-42* by *set2Δ* (Figure 5C). This finding reveals a cryptic positive regulatory role of Set3 on Nab3, potentially explained through its role in promoting the accumulation of Nab3-K363me1. Curiously, the magnitude of this cryptic *SET3* activating function was dampened in a *nab3-42 set1Δ set3Δ* mutant (Figure 5C). These findings suggest that potential regulatory functions of Nab3-K363me1 are subtle and manifested in a complex manner, contributing to an increasingly sophisticated network of NNS regulation by *SET1*, *SET2* and *SET3* (Figure 6 and discussed below).

## DISCUSSION

Post-translational modifications (PTMs) are well known for their ability to impart protein regulation through a diverse array of molecular mechanisms. Although protein lysine methylation is most prominently understood through studies of histone methylation, this modification has emerged as a PTM that impacts a wide array of proteins (60). Belying the known recruitment of numerous histone lysine methyltransferases to chromatin, the identification of methyllysines in chromatin-associated proteins is still nascent. We identify here nine methylated lysine residues in the NNS transcriptional termination complex and show that one of these residues, Nab3-K363, is of critical importance for NNS function through its role in mediating RNA binding. We find that mono-methylation of Nab3-K363 is strongly reduced in strains lacking *SET1*, a known regulator of NNS function. Moreover, Nab3-K363me1 is essentially abolished in strains lacking *SET3*, a target of Set1 activation through H3K4me2 (23,24,40). While our findings suggest numerous regulatory scenarios by which *SET1*/*3* control Nab3-K363me1, the simplest posits a pathway model in which Set3 directly methylates Nab3-K363 in response to activation by Set1-deposited H3K4me2 (Figure 6).

Of the nine NNS methylated lysine residues we identified, only Nab3-K363 exhibited an obvious functional role. Our studies are not sufficient to rule out more subtle functional roles for the eight additional methylation sites, and more detailed interrogation will be needed to address this. One intriguing possibility is that methylation of these residues by one or more enzymes act in concert to regulate NNS function. It will thus be interesting to determine if *SET1/3* control these other NNS modifications. Our studies of Nab3-K363 have homed in on RNA binding as the critical function for this residue, and structural studies have revealed that Nab3-K363 forms a hydrogen bond with the RNA backbone of a target RNA (Figure 4A) (37,38). The RNA binding properties of wild-type Nab3-K363, Nab3-K363A and Nab3-K363R reveal that the precise biochemical characteristics of Nab3-K363 are crucial for Nab3 RNA binding and NNS function. While this is not surprising given that Nab3-K363 forms a hydrogen bond with the RNA backbone, they do suggest that the methylation of Nab3-K363 may alter its binding, perhaps through charge shielding or altering the hydrophobic character and/or size of the modified lysine. As much of our findings rely on investigation of Nab3-K363R, which does not precisely mimic Nab3-K363me0, detailed studies of the biochemical and biophysical consequences of mono-methylation of Nab3-K363 will be necessary to comprehend the regulatory impact of this PTM.

We found that ∼75% of Nab3-K363me1 depends on the presence of *SET1* (Figure 5A and B). A possible explanation for this finding is that Set1, along with at least one additional methyltransferase, directly methylates Nab3-K363. Of course, it is also easy to envision indirect control of Nab3-K363 methylation by *SET1*. The most plausible such indirect mechanism is through Set1’s control of H3K4 methylation, which in turn mediates the recruitment/regulation of numerous other chromatin modifying factors. Paramount among these is the Set3 HDAC, which is positively regulated through a specific interaction of Set3 with H3K4me2 (40). We report that Nab3-K363me1 is essentially abolished in a *set3Δ* strain. Set3 contains a SET domain, which may possess some yet-to-be-identified lysine methyltransferase activity. As mentioned above, a parsimonious model explaining these findings posits that Set3 directly methylates Nab3-K363, and that Set1 provides an activating role in this via H3K4me2 (Figure 6). Alternatively, Set1 and Set3 may each directly methylate Nab3-K363, separately controlling a possible regional accumulation of Nab3-K363me1 across the chromatin landscape.

In the course of our investigations, we discovered that *SET2* exerts a strong repressive impact on *NAB3* via H3K36 methylation. This role for H3K36 methylation contributes to an increasingly complex nexus of NNS regulation involving *SET1*, *SET3*, H3K4 methylation, and the RNA PolII CTD phosphorylation state (Figure 6). How might Nab3-K363me1 factor into this nexus? We report that when *SET2* function is absent, a cryptic *NAB3* activating role for *SET3* is apparent (Figure 5C). As Nab3-K363me1 is essentially absent in *set3Δ*, a potential activating role for Nab3-K363me1 may explain these genetic interaction results. It is noteworthy that the activating role for *SET3* is only observed in *nab3-42 set2Δ* double mutants, suggesting that any activating function of Nab3-K363me1 is subtle. A contrasting, repressive role of Nab3-K363me1 may be hypothesized based on our *SET1* genetic findings. However, unlike *set3Δ*, *set1Δ*, which also caused a large reduction in Nab3-K363me1, resulted in enhanced *NAB3* activity in a manner independent of H3K4 methylation (Figure 5A–C) (24). Lending credence to the possibility that regionally deposited Nab3-K363me1 by Set1 and Set3 exert differing consequences on *NAB3*, *set1Δ* and *set3Δ* exhibited additive effects when combined with *nab3-42 set2Δ* (Figure 5C). In addition to determining the biochemical consequences of Nab3-K363me1, it will thus be critical to deduce the distribution of Nab3-K363me1 across the genomic landscape and the roles of Set1 and Set3 in controlling this.

Numerous yeast studies have identified methylated lysine and arginine residues on yeast proteins, including some that are chromatin associated (61–63). Our interrogation of NNS reported here identifies numerous lysine methylations. An exciting possibility envisioned by these findings is that lysine methylation of chromatin-associated proteins is common, and that some of these may be of regulatory significance. Indeed, although numerous histone methyltransferases have been highly characterized with respect to their ability to control methylation at specific histone residues, the known targets of these enzymes remain largely restricted to their respective histone substrates. Whether the regulatory impact of these methyltransferases is explained solely through their histone targets remains an open question.

## DATA AVAILABILITY

All data is available upon request.

## Supplementary Material

gkaa029_Supplemental_Files
