# Supplementary material for: A crucial RNA-binding lysine residue in the Nab3 RRM domain undergoes SET1 and SET3-responsive methylation
Source: Nucleic Acids Res. 2020 Jan 21;48(6):2897–911. doi: 10.1093/nar/gkaa029 (PMC7102954; doi:10.1093/nar/gkaa029)
Supplement: gkaa029_Supplemental_Files [file gkaa029_supplemental_files.zip › Nab3_paper_suppl_figures_.pdf]

Figure S1

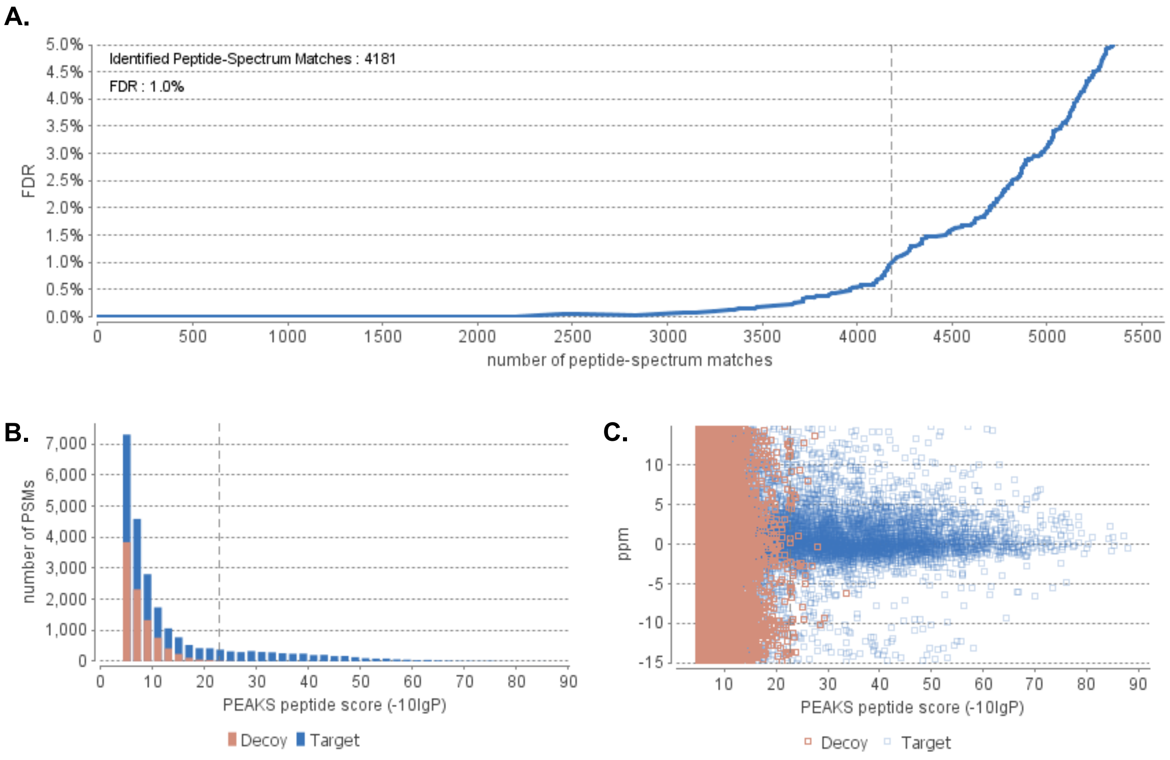

Figure S1. Analysis of false discovery rates used for Kme peptide identification using target-decoy. (A) False discovery rate (FDR) curve. X-axis is the number of peptide-spectrum matches (PSM) being kept. Y-axis is the corresponding FDR. The vertical dashed line represents the 1.0% FDR cut-off. (B) Distribution of PEAKS peptide score (-10lgP) compared to decoy identification (C) Scatterplot of PEAKS peptide score versus precursor mass error. The vertical dashed line represents the 1.0% FDR cut-off.

Figure S2

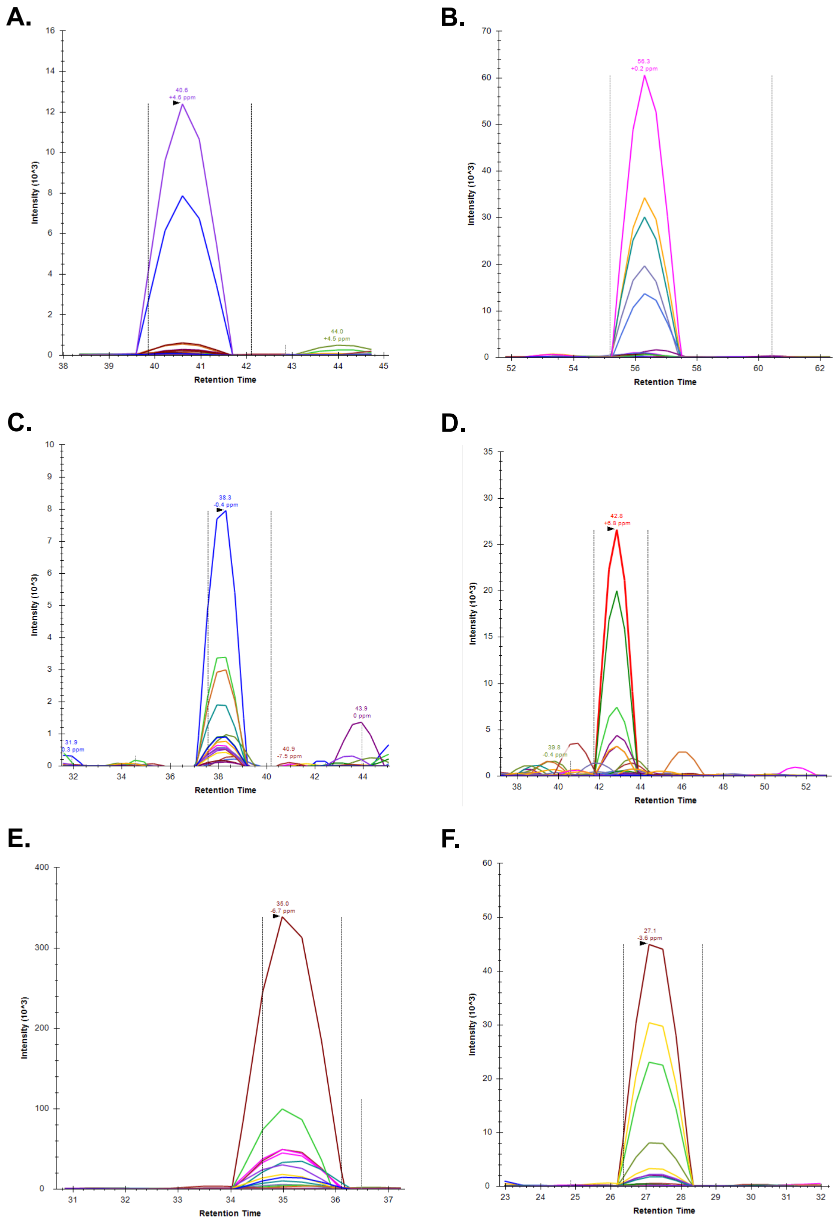

Figure S2

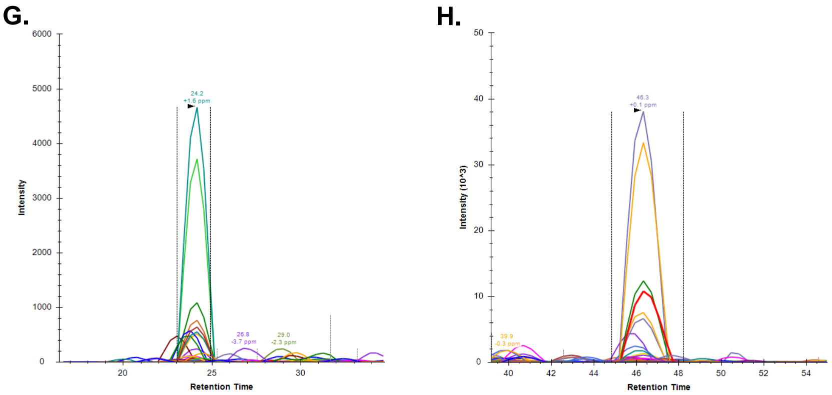

Figure S2. SRM-MS based identification of novel Nab3, and Sen1 lysine methylation events. Transition ions for (A) Nab3(K73me1), (B) Nab3(K213me1), (C) Nab3(K363me3), (D) Nab3(K393me2), (E) Sen1(K19me1), (F) Sen1(K19me2), (G) Sen1(K21me1), and (H) Sen1(K21me2) methylations are shown. Chromatographs were compiled by Skyline v.2.5.0.5675 software.

Figure S3

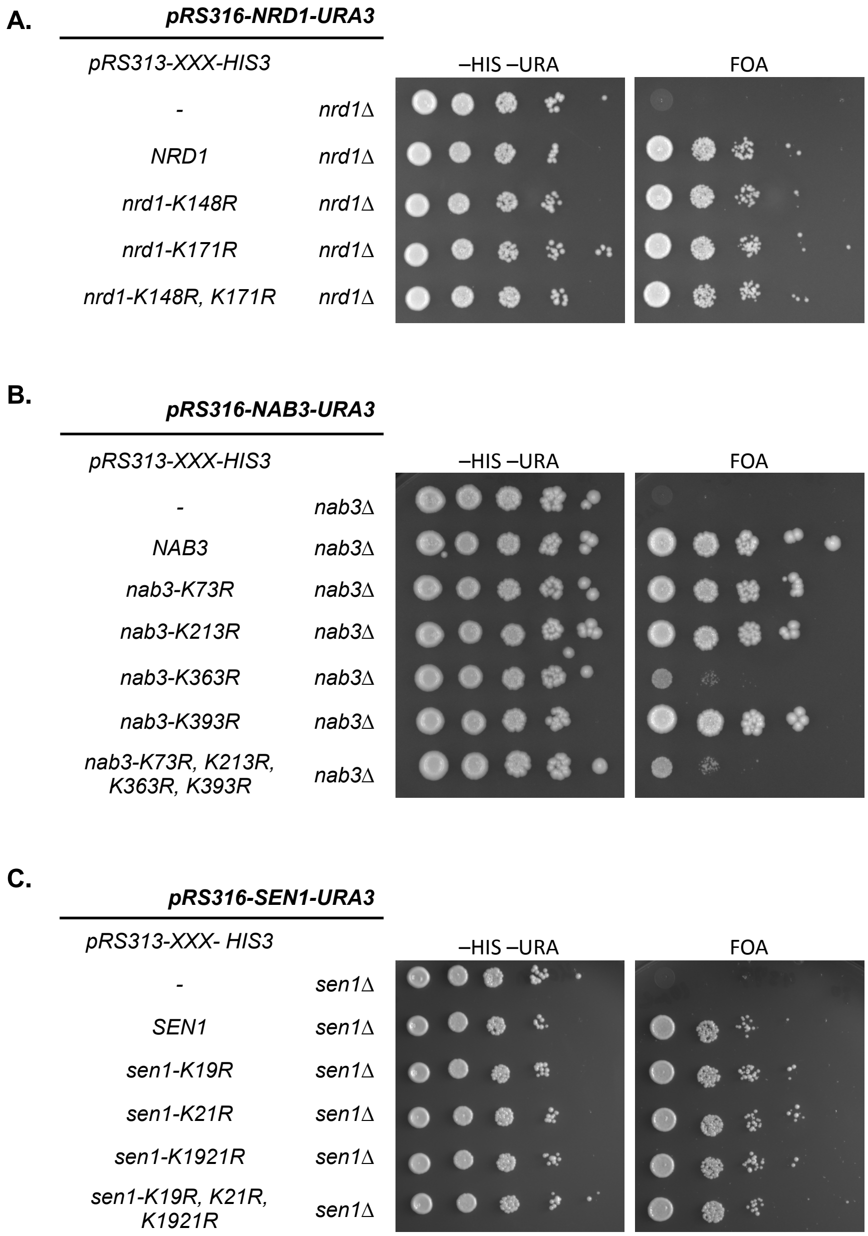

Figure S3. Systematic mutation of methylated lysine residues to arginine. Yeast strains possessing the indicated plasmids were serially diluted ten-fold, spotted onto agar plates containing -HIS -URA dropout media and also on synthetic complete media supplemented with 5FOA. The “-” symbol represents pRS313-HIS3 (empty vector). All strains were grown at 30°C. Systematic mutation of (A) Nab3, (B) Nrd1, (C) Sen1 methylated lysine residues to arginine.

Figure S4

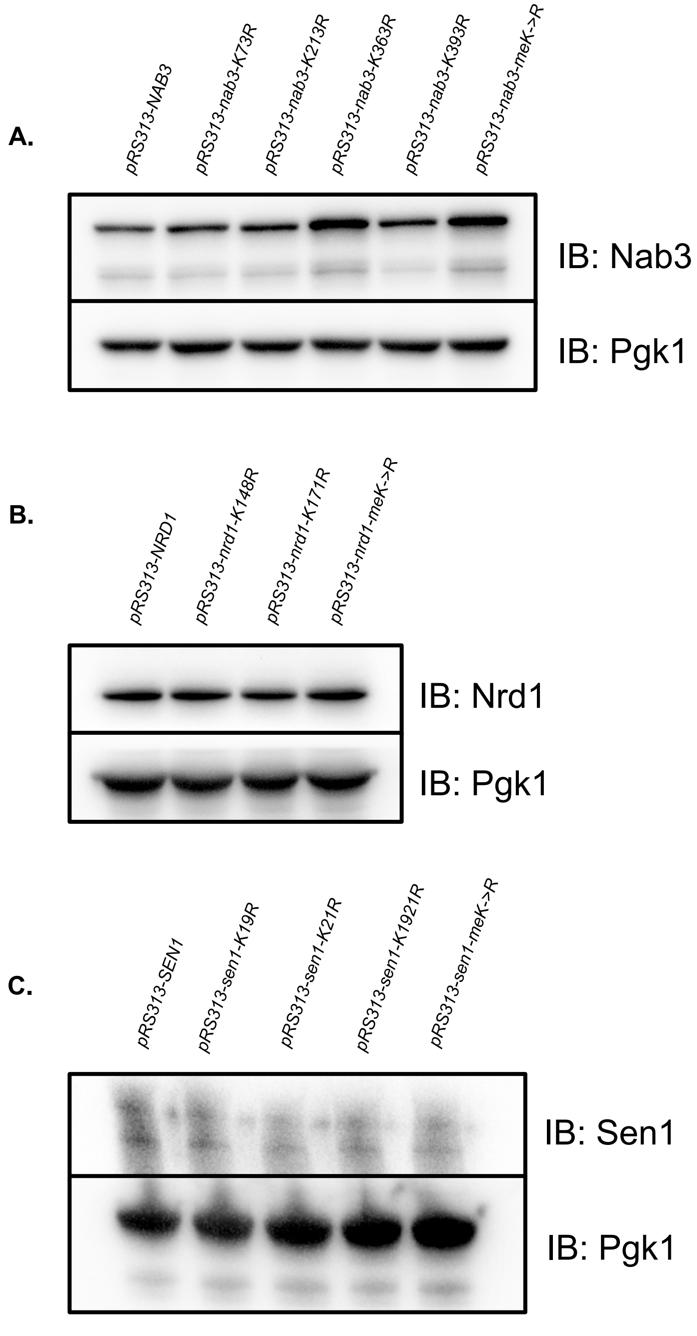

Figure S4. Nrd1, Nab3, Sen1 K to R mutants all express stable proteins. Immunoblot analysis of (A) Nab3, (B) Nrd1, and (C) Sen1 protein levels in wildtype and respective lysine to arginine substitution mutants. meK→R represents mutants with all methylated lysine residues mutated to arginine.

Figure S5

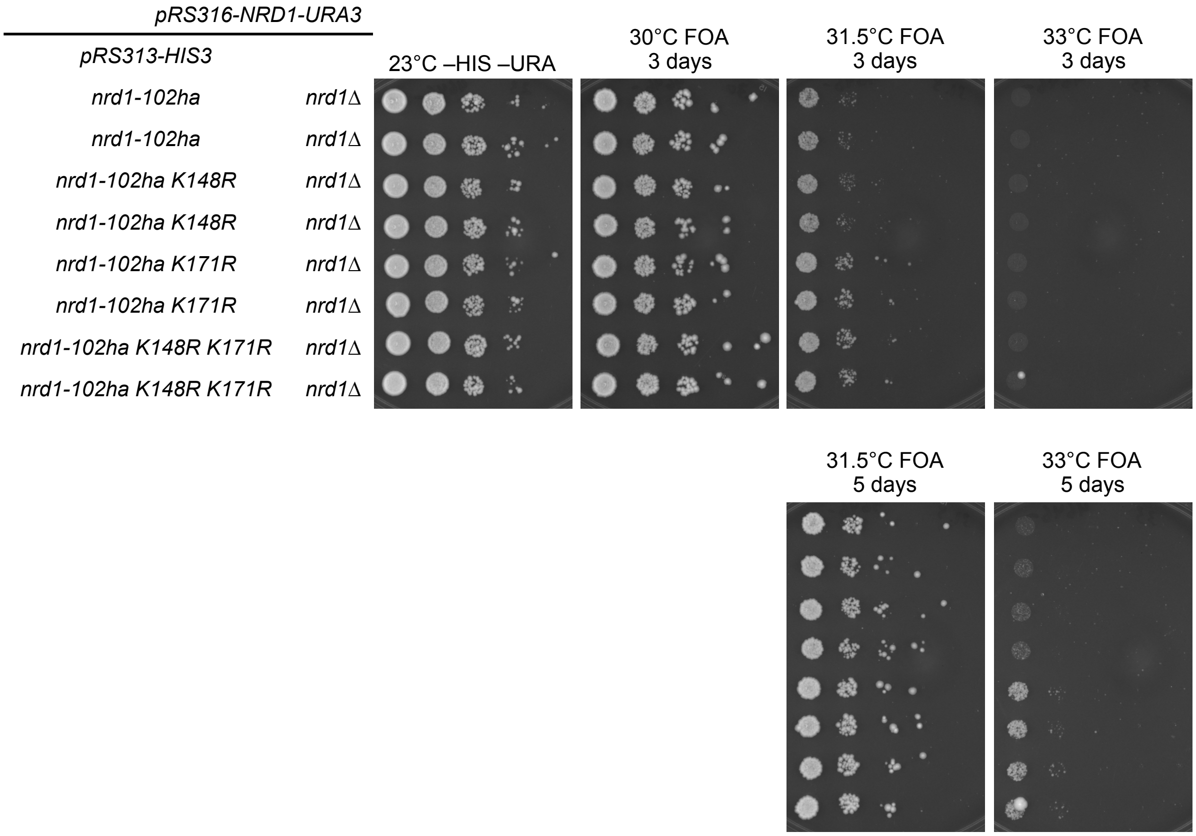

Figure S5. *nrd1-102ha* mutants are weakly suppressed by a K171 to arginine substitution. Yeast strains possessing the indicated plasmids were serially diluted ten-fold, spotted onto agar plates containing -HIS -URA dropout media and also on synthetic complete media supplemented with 5FOA. Growth of two independent isolates of each genotype is shown. Plates at 23°C -HIS-URA and 30°C FOA are shown after 3 days of growth, while plates at 31.5°C, and 33°C FOA are shown after 3 days and 5 days of growth.

Figure S6

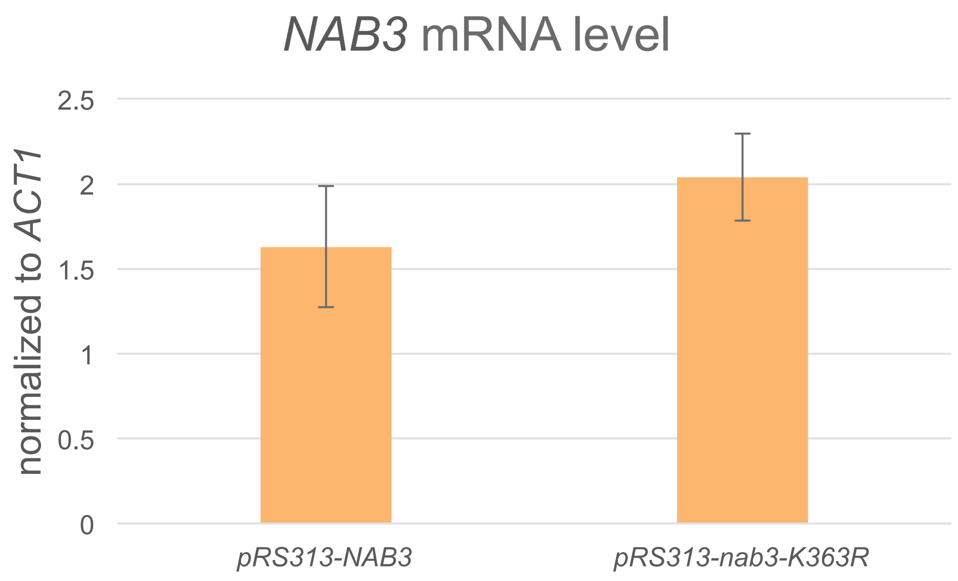

Figure S6. Steady state level of the *NAB3* mRNAs. *NAB3* mRNA level from *NAB3* and *nab3-K363R* cells was analyzed by qRT-PCR. The average of three biological replicates is shown. Error bars represent standard deviation. Significance is calculated by a two-tailed student's t-test $p>0.05$, $n=3$.

Figure S7

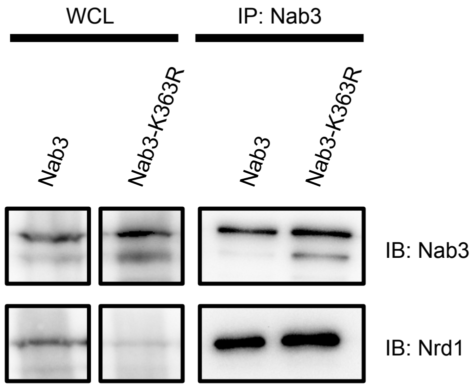

Figure S7. Nab3 immunoprecipitation in wildtype and *nab3-K363R* mutants. Total cell lysates (WCL) were immunoprecipitated with a monoclonal Nab3 antibody (IP: Nab3) followed by immunoblot analysis with anti-Nab3 and anti-Nrd1 antibodies.

Figure S8

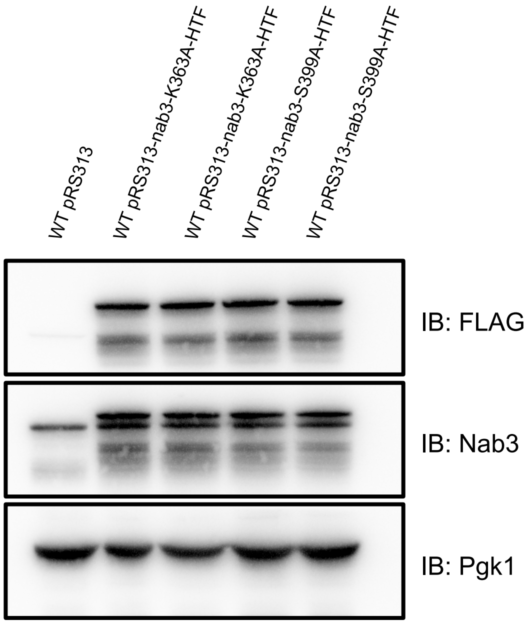

Figure S8. Nab3-K363A and Nab3-S399A plasmid constructs express stable proteins. Immunoblot analysis of wildtype cells expressing *pRS313* (empty vector), *pRS313-nab3-K363A-HTF*, and *pRS313-nab3-S399A-HTF*. Nab3-K363A-HTF (His_6-TEV-FLAG) and Nab3-S399A-HTF protein were detected by an anti-FLAG antibody as well as the anti-Nab3 antibody. On the Nab3 blot, the top band likely represents the Nab3-HTF construct while the bottom band represents endogenous Nab3. Pgk1 protein level was detected using the anti-Pgk1 antibody and used as a loading control.

Figure S9

A.

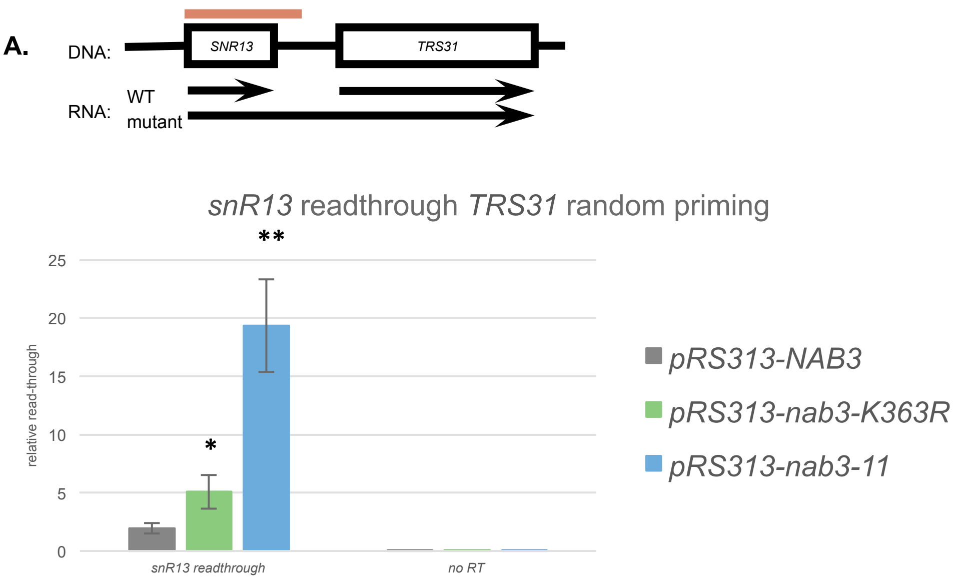

B.

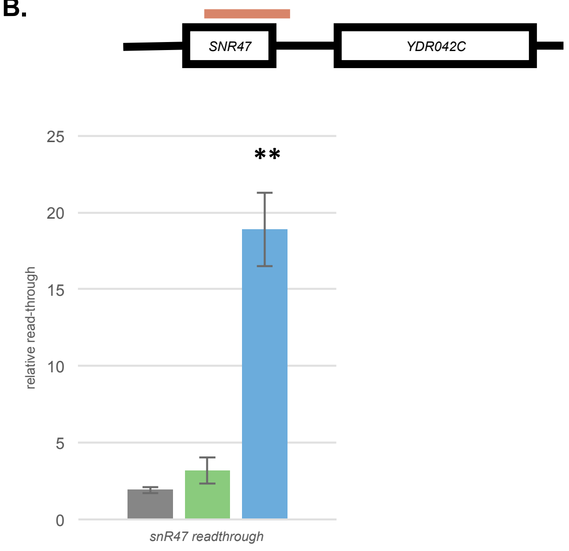

Figure S9. qRT-PCR analysis of *NAB3*, *nab3-K363R*, and *nab3-11* cells. (A) *TRS31* random priming matches the results seen for gene specific priming. Total RNA from *NAB3, nab3-K363R,* and *nab3-11* cells was processed into cDNA by random nonamers instead of a *TRS31* gene specific primer. The presence of *SNR13* read-through transcription was analyzed by qPCR. (B) The level of read-through transcription at *SNR47*. Error bars represent standard deviation of three biological replicates. Significance between each mutant and the WT control is calculated by a two-tailed student's t-test and denoted by * $p<0.05$, ** $p<0.01$, n=3. No reverse transcription control (No RT, n=1).

Table S10. Relative fold-change in methylation status of select Nab3 and Sen1 lysine methylation sites. Methylation status was monitored by PRM-MS and determined by total transition peak area. Log2 fold-change values are relative to wild-type (WT) strain.

		set1Δ	*set2Δ*	*set3Δ*
Nab3	K73me1			
	**K363me1**			
	K393me2			
Sen1	K19me1			
	K21me1			
	K21me2			
	K1921me1			

Relative peak area (Log2 fold-change)

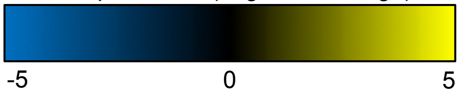

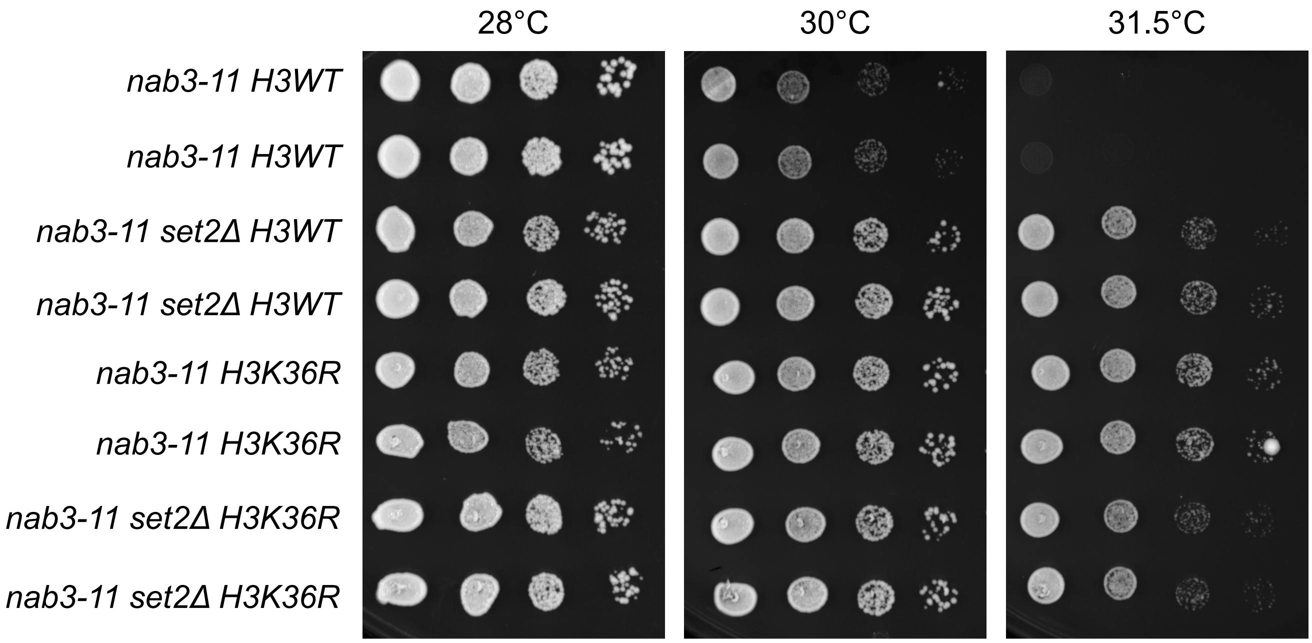

Figure S11. Genetic interactions of *nab3-11* with *set2*Δ and H3K36R. Yeast strains of the indicated genotypes were serially diluted ten-fold, spotted onto agar plates, and grown at the specified temperatures.
