# Supplementary material for: A crucial RNA-binding lysine residue in the Nab3 RRM domain undergoes SET1 and SET3-responsive methylation
Source: Nucleic Acids Res. 2020 Jan 21;48(6):2897–911. doi: 10.1093/nar/gkaa029 (PMC7102954; doi:10.1093/nar/gkaa029)
Supplement: gkaa029_Supplemental_Files [file gkaa029_supplemental_files.zip › Table S1^3.docx]

| **Strain** | **Mating Type** | **Genotype** | **Plasmid-1** | **Plasmid-2** |
| --- | --- | --- | --- | --- |
|  |  |  |  |  |
| kly2172 | Mat**α** | his3 leu2 ura3 |  |  |
|  |  | congenic to BY4742 |  |  |
|  |  |  |  |  |
|  |  | congenic to kly2172 |  |  |
| kly4289 | Mat**a** | nab3∆::kanMX4 | pRS313-NAB3-HIS3 |  |
| kly4291 | Mat**a** | nab3∆::kanMX4 | pRS313-nab3-K363R-HIS3 |  |
| kly4528 | Mat**a** | sen1∆::kanMX4 | pRS313-SEN1-HIS3 |  |
| kly4529 | Mat**a** | sen1∆::kanMX4 | pRS313-sen1-K19R-HIS3 |  |
| kly4530 | Mat**a** | sen1∆::kanMX4 | pRS313-sen1-K21R-HIS3 |  |
| kly4531 | Mat**a** | sen1∆::kanMX4 | pRS313-sen1-K1921R-HIS3 |  |
| kly4534 | Mat**a** | nrd1∆::kanMX4 | pRS316-NRD1-URA3 | pRS313-HIS3 |
| kly4535 | Mat**a** | nrd1∆::kanMX4 | pRS316-NRD1-URA3 | pRS313-NRD1-HIS3 |
| kly4536 | Mat**a** | nrd1∆::kanMX4 | pRS316-NRD1-URA3 | pRS313-nrd1-K148R-HIS3 |
| kly4537 | Mat**a** | nrd1∆::kanMX4 | pRS316-NRD1-URA3 | pRS313-nrd1-K171R-HIS3 |
| kly4538 | Mat**a** | nrd1∆::kanMX4 | pRS316-NRD1-URA3 | pRS313-nrd1-2K->R-HIS3 |
| kly4546 | Mat**a** | sen1∆::kanMX4 | pRS316-SEN1-URA3 | pRS313-HIS3 |
| kly4547 | Mat**a** | sen1∆::kanMX4 | pRS316-SEN1-URA3 | pRS313-SEN1-HIS3 |
| kly4548 | Mat**a** | sen1∆::kanMX4 | pRS316-SEN1-URA3 | pRS313-sen1-K19R-HIS3 |
| kly4549 | Mat**a** | sen1∆::kanMX4 | pRS316-SEN1-URA3 | pRS313-sen1-K21R-HIS3 |
| kly4550 | Mat**a** | sen1∆::kanMX4 | pRS316-SEN1-URA3 | pRS313-sen1-K1921R-HIS3 |
| kly4566 | Mat**a** | nab3∆::kanMX4 | pRS313-nab3-11-HIS3 |  |
| kly4568 | Mat**α** | nab3∆::kanMX4 | pRS316-NAB3-URA3 | pRS313-HIS3 |
| kly4569 | Mat**α** | nab3∆::kanMX4 | pRS316-NAB3-URA3 | pRS313-NAB3-HIS3 |
| kly4570 | Mat**α** | nab3∆::kanMX4 | pRS316-NAB3-URA3 | pRS313-nab3-K73R-HIS3 |
| kly4571 | Mat**α** | nab3∆::kanMX4 | pRS316-NAB3-URA3 | pRS313-nab3-K213R-HIS3 |
| kly4572 | Mat**α** | nab3∆::kanMX4 | pRS316-NAB3-URA3 | pRS313-nab3-K363R-HIS3 |
| kly4573 | Mat**α** | nab3∆::kanMX4 | pRS316-NAB3-URA3 | pRS313-nab3-K393R-HIS3 |
| kly4574 | Mat**α** | nab3∆::kanMX4 | pRS316-NAB3-URA3 | pRS313-nab3-4K->R-HIS3 |
| kly4577 | Mat**a** | sen1∆::kanMX4 | pRS316-SEN1-URA3 | pRS313-sen1-3K->R-HIS3 |
| kly4580 | Mat**α** | nab3∆::kanMX4 | pRS316-NAB3-URA3 | pRS313-nab3-K363A-HIS3 |
| kly4612 | Mat**α** | nab3∆::kanMX4 | pRS313-NAB3-HIS3 |  |
| kly4614 | Mat**α** | nab3∆::kanMX4 | pRS313-nab3-K73R-HIS3 |  |
| kly4616 | Mat**α** | nab3∆::kanMX4 | pRS313-nab3-K213R-HIS3 |  |
| kly4618 | Mat**α** | nab3∆::kanMX4 | pRS313-nab3-K393R-HIS3 |  |
| kly4643 | Mat**α** | nab3∆::kanMX4 | pRS313-nab3-K363R-HIS3 |  |
| kly4644 | Mat**α** | nab3∆::kanMX4 | pRS313-nab3-4K->R-HIS3 |  |
| kly4646 | Mat**α** | nrd1∆::kanMX4 | pRS316-NRD1-URA3 | pRS313-nrd1-102ha-HIS3-6 |
| kly4647 | Mat**α** | nrd1∆::kanMX4 | pRS316-NRD1-URA3 | pRS313-nrd1-102ha-HIS3-8 |
| kly4648 | Mat**α** | nrd1∆::kanMX4 | pRS316-NRD1-URA3 | pRS313-nrd1-102ha-K148R-HIS3-3 |
| kly4649 | Mat**α** | nrd1∆::kanMX4 | pRS316-NRD1-URA3 | pRS313-nrd1-102ha-K148R-HIS3-4 |
| kly4650 | Mat**α** | nrd1∆::kanMX4 | pRS316-NRD1-URA3 | pRS313-nrd1-102ha-K171R-HIS3-2 |
| kly4651 | Mat**α** | nrd1∆::kanMX4 | pRS316-NRD1-URA3 | pRS313-nrd1-102ha-K171R-HIS3-3 |
| kly4652 | Mat**α** | nrd1∆::kanMX4 | pRS316-NRD1-URA3 | pRS313-nrd1-102ha-2K->R-HIS3-1 |
| kly4653 | Mat**α** | nrd1∆::kanMX4 | pRS316-NRD1-URA3 | pRS313-nrd1-102ha-2K->R-HIS3-2 |
| kly4676 | Mat**a** | nrd1∆::kanMX4 | pRS313-NRD1-HIS3 |  |
| kly4702 | Mat**a** | sen1∆::kanMX4 | pRS313-sen1-3K->R-HIS3 |  |
| kly4703 | Mat**a** | nrd1∆::kanMX4 | pRS313-nrd1-K148R-HIS3 |  |
| kly4704 | Mat**a** | nrd1∆::kanMX4 | pRS313-nrd1-K171R-HIS3 |  |
| kly4705 | Mat**a** | nrd1∆::kanMX4 | pRS313-nrd1-2K->R-HIS3 |  |
| kly4750 | Mat**α** | nab3∆::kanMX4 | pRS316-NAB3-URA3 | pRS313-nab3-S399A-HIS3 |
| kly4773 | Mat**a** | WT | pRS313-HIS3 |  |
| kly4781 | Mat**a** | WT | pRS313-nab3-K363A-HTF-HIS3-1 | |
| kly4782 | Mat**a** | WT | pRS313-nab3-K363A-HTF-HIS3-3 | |
| kly4783 | Mat**a** | WT | pRS313-nab3-S399A-HTF-HIS3-3 | |
| kly4784 | Mat**a** | WT | pRS313-nab3-S399A-HTF-HIS3-4 | |
| mmy6362 | Mat**a** | NRD1-TAP::HIS3 |  |  |
| mmy6363 | Mat**a** | SEN1-TAP::HIS3 |  |  |
| mmy6364 | Mat**a** | NAB3-TAP::HIS3 |  |  |
| kly3776 | Mat**α** | NAB3-TAP::HIS3 set1∆::hygMX6 | |  |
| mmy7440 | Mat**a** | NAB3-TAP::HIS3 set2∆::KanMX6 | |  |
| mmy7443 | Mat**a** | NAB3-TAP::HIS3 set3∆::KanMX6 | |  |
| kly4624 | Mat**a** | WT | |  |
| kly4625 | Mat**a** | set3∆::kanMX4 | |  |
| kly4626 | Mat**a** | set1∆::natMX6 | |  |
| kly4627 | Mat**α** | set2∆::hygMX6 | |  |
| kly4628 | Mat**α** | set2∆::hygMX6 set3∆::kanMX4 | |  |
| kly4629 | Mat**α** | set1∆::natMX6 set3∆::kanMX4 | |  |
| kly4630 | Mat**α** | set1∆::natMX6 set2∆::hygMX6 | |  |
| kly4631 | Mat**a** | set1∆::natMX6 set2∆::hygMX6 set3∆::kanMX4 | |  |
| kly4632 | Mat**a** | nab3-42::URA3 | |  |
| kly4633 | Mat**a** | nab3-42::URA3 set3∆::kanMX4 | |  |
| kly4634 | Mat**α** | nab3-42::URA3 set1∆::natMX6 | |  |
| kly4553 | Mat**a** | nab3-42::URA3 set2∆::hygMX6 | |  |
| kly4635 | Mat**a** | nab3-42::URA3 set2∆::hygMX6 set3∆::kanMX4 | |  |
| kly4636 | Mat**a** | nab3-42::URA3 set1∆::natMX6 set3∆::kanMX4 | |  |
| kly4637 | Mat**a** | nab3-42::URA3 set1∆::natMX6 set2∆::hygMX6 | |  |
| kly4638 | Mat**a** | nab3-42::URA3 set1∆::natMX6 set2∆::hygMX6 set3∆::kanMX4 | |  |
| kly3926 | Mat**a** | nab3-11::kanMX4 hht1-hhf1∆::natMX4 hht2-hhf2::[HHTS-HHFS]H3WT-URA3 | |  |
| kly3927 | Mat**α** | nab3-11::kanMX4 hht1-hhf1∆::natMX4 hht2-hhf2::[HHTS-HHFS]H3WT-URA3 | |  |
| kly3928 | Mat**a** | nab3-11::kanMX4 hht1-hhf1∆::natMX4 hht2-hhf2::[HHTS-HHFS]H3WT-URA3 set2∆::hygMX6 | |  |
| kly3929 | Mat**α** | nab3-11::kanMX4 hht1-hhf1∆::natMX4 hht2-hhf2::[HHTS-HHFS]H3WT-URA3 set2∆::hygMX6 | |  |
| kly3930 | Mat**α** | nab3-11::kanMX4 hht1-hhf1∆::natMX4 hht2-hhf2::[HHTS-HHFS]H3K36R-URA3 | |  |
| kly3931 | Mat**α** | nab3-11::kanMX4 hht1-hhf1∆::natMX4 hht2-hhf2::[HHTS-HHFS]H3K36R-URA3 | |  |
| kly3932 | Mat**a** | nab3-11::kanMX4 hht1-hhf1∆::natMX4 hht2-hhf2::[HHTS-HHFS]H3K36R-URA3 set2∆::hygMX6 | |  |
| kly3933 | Mat**a** | nab3-11::kanMX4 hht1-hhf1∆::natMX4 hht2-hhf2::[HHTS-HHFS]H3K36R-URA3 set2∆::hygMX6 | |  |
