# Supplementary material for: A crucial RNA-binding lysine residue in the Nab3 RRM domain undergoes SET1 and SET3-responsive methylation
Source: Nucleic Acids Res. 2020 Jan 21;48(6):2897–911. doi: 10.1093/nar/gkaa029 (PMC7102954; doi:10.1093/nar/gkaa029)
Supplement: gkaa029_Supplemental_Files [file gkaa029_supplemental_files.zip › Table S2.pdf]

Table S2

Primer Name	Sequence (5' -> 3')
TRS31_847R (Used for Reverse Transcription)	CAAATCTCAAACCTTCCCTG
sNR13_F	AGGAAGTTTTTTCCTTTT
sNR13_113downstream_R	CCAAACCCAACGTACTAAC
ACT1-Fwd1	TGTCACCAACTGGGACGATA
ACT1-Rev1	AACCAGCGTAAATTGGAACG
NAB3_ORF_F	CTACAAAGGCTCACAAGG
NAB3_ORF_R	ATAGCACGGTAATCAGTAGG
snR5_HEM4_1F	GCAAATGGCTGGAAGTAG
snR5_HEM4_1R	CTTAGCTGACTACAGCAC
snR33_CTO1_1F	CAGAGATTGAAAGCCTAGC
snR33_CTO1_1R	CTCGATTGTCAACATTGCT
snR47_YDR042C_1F	ACATGAATTTCTTCGTCCGA
snR47_YDR042C_1R	GGAGGATGAAGCACAATAAG
snR48_ERG25_2F	GTGAAGTTTAAGTACTCTCC
snR48_ERG25_2R	GGGTAACGAATGGATTGC
NEL025C up	GTAGTCGCCAACAATCATTTCGATACAACTTG
NEL025C down	CAACCGCTGTTGTCAAACAAGACTATAGG
snR47 oligo for RNA binding assays	[Btn]UUUCUUUUCUUAUUCUUAUU
